# Proposal of Molecular‐Level Crystallization Mechanism for Halogenated Benzyl Alcohols: A Study of Isostructural Crystals

**DOI:** 10.1002/cplu.202500144

**Published:** 2025-06-20

**Authors:** Patrick Teixeira Campos, Pedro Henrique Cunha do Couto, Álex Canez Lemos Souza, Juliano Alex Roehrs

**Affiliations:** ^1^ Laboratório de Química Orgânica Sintética Estrutural e Computacional (LaQuiOSEC) Instituto Federal de Educação Ciência e Tecnologia Sul‐rio‐grandense (IFSul) – Câmpus Pelotas Pelotas CEP 96015‐360 Brazil

**Keywords:** crystal engineering, density functional theory, halogen bonding, intermolecular interactions, self‐assembly

## Abstract

Herein, crystallization mechanisms for halogenated benzyl alcohols, based on theoretical energetic and topological data of intermolecular and supramolecular interactions, are proposed. All interactions are verified and energetically classified by density functional theory and their contribution at each contact point is analyzed using quantum theory of atoms in molecules model. Weak interactions, such as C—H···X, C—H···C e C···X, perform a key role in stabilizing the crystal packing due to the larger contact area of these fragments and their higher occurrence, compared to strong interactions such as O—H···O. The crystallization mechanisms are proposed based on the model developed by the research group. The mechanism starts with the strongest interaction (π···π) present in the first coordination sphere, which forms the first 1D supramolecular chain. From this chain, hypotheses of approximation between these chains are analyzed to determine the most stable arrangement for the formation of the supramolecular layer 2D. The process continues until the formation of a supramolecular structure (3D) with growth in its three directions through interactions X···X via σ‐hole. The compounds in the *ortho* and *para* positions demonstrated isostructuralism in the equivalent positions, even with the variation of the halogen (chlorine and bromine).

## Graphical abstract

Proposals for crystallization mechanisms for halogenated benzyl alcohols at the molecular level show that strong interactions such as OH···O and π···π initiate the crystallization process, although weak interactions (C—H···X, C—H···C, and X···X) are crucial for stabilizing crystal packing. The study also shows how isostructural compounds, with halogen variation in equivalent positions, have different crystallization mechanisms.

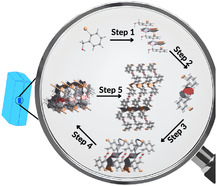

## 1 Introduction

Solution crystallization is a unit operation commonly used in various industries^[^ ^1^ ^]^ to obtain a pure final product^[^ ^2^ ^]^ and with the desired properties. Crystalline properties permeate several areas such as: pharmaceuticals, information technology, and food, and in this universe, understanding the crystallization mechanism is of utmost importance to determine a correlation between structure and property.^[^ ^3^ ^]^ In this context, crystal engineering emerges as a topic of great popularity in the scientific community, with several studies highlighting the importance of intermolecular interactions in determining crystalline properties.^[^ ^4^ ^]^ Recent research has established a relationship between mechanical properties and halogen bonding via σ‐hole, highlighting the crucial role of halogen modification in the observed properties. In particular, strong halogen bonds are associated with elastic deformation, while weaker bonds tend to result in plastic bending.^[^ ^4^ ^]^ Similarly, benzyl esters with alcoholic or phenolic groups have different properties, in which the formation of stronger hydrogen bonds (resulting from the alcohol hydroxyl) favors the formation of an elastic structure.^[^ ^5^ ^]^ Flexible organic crystals, whether elastic or irreversibly bendable plastics, are of great interest due to their wide application in lithium batteries,^[^ ^6^ ^]^ solar panels,^[^ ^6^ ^]^ optical devices,^[^ ^7^ ^]^ and muscle mimetic biomaterials.^[^ ^8^ ^]^ Although, the understanding that intermolecular interactions directly influence mechanical properties, it is still not known how and when these interactions are formed at the molecular level during crystallization. Nucleation plays an important role in the initiation of crystallization, determining the polymorphic form of the crystalline phase.^[^ ^9^ ^]^ Currently the review described by Barua et al.^[^ ^10^ ^]^ reports three main theories well established in the scientific community, and classical, two‐step nucleation and prenucleation, which are exhaustively discussed in the literature.

The systematic investigation of intermolecular interactions can be conducted effectively through the selective modification of atoms in a series of compounds,^[^ ^11^ ^]^ a strategy that favors obtaining isostructural crystals with different chemical compositions. Analysis of these crystals allows an in‐depth assessment of crystal packing, providing insights into the structure's ability to tolerate molecular modifications without significant changes to its 3D organization.^[^ ^12^ ^]^

Intermolecular interactions motivate self‐assembly in the crystallization process.^[^ ^13^ ^]^ A topological approach assists to understand this process of self‐assembly and self‐organization, as it establishes that the approximation of molecular fragments occurs via noncovalent bonds in energetically favorable areas.^[^ ^14^ ^]^ Computational calculations have proven to be an important tool for energetic^[^ ^15^, ^16^ ^]^ and topological analysis^[^ ^16^ ^]^ to determine the energy hierarchy in the interactions present in the cluster and thus suggest crystallization mechanisms.^[^ ^14^ ^]^

Thus, density functional theory (DFT) with dispersion correction (D3) is an important accurate method for estimating the energy of intermolecular interactions,^[^ ^17^ ^]^ frequently used in developing proposals for nucleation mechanisms at the molecular level.^[^ ^18^ ^]^ Previously our research group published studies suggesting crystallization mechanisms for phenols,^[^ ^19^ ^]^ benzamides,^[^ ^20^ ^]^ and benzoic acids,^[^ ^21^ ^]^ all halogenated. In these works, energetic analysis of the intermolecular interactions found in the first coordination sphere were carried out, to determine the supramolecular growth in a given direction. This evaluation was carried out until growth was evident in all three directions and consequently demonstrating all interactions observed in the cluster, as already described in the literature.^[^ ^22^ ^]^

However, the study of the first coordination sphere does not seem to be sufficient to determine the number of crystallization steps. According to the nonclassical theory, during crystallization there is an approximation of intermediate supramolecular chains, which are not considered in the study of the cluster previously proposed. Thereby, the evaluation of the approximation between supramolecular chains becomes important, since the sum of weaker interactions can sometimes overcome stronger interactions. Moreover, this model does not predict a possible growth along a direction already observed before the completion of the proposed mechanism. With this in mind, our group developed a new theoretical model that employs a rational and systematic strategy based on the determination of the energy of supramolecular interactions, not only between chains (1D) but also between layers (2D).^[^ ^22^ ^]^ Furthermore, recently Biran et al.^[^ ^23^ ^]^ experimentally describes the nucleation mechanism of perylene diimide that occurs through self‐assembly and follows a hierarchy of interactions on a molecular scale to form disordered stacks. These stacks stiffen to interact with each other and form a crystalline domain that merges with each other to form the precipitated crystal. Another recent experimental study described by Elizabeth et al.^[^ ^24^ ^]^ corroborates with the approach that states that the crystallization of coronene dipeptide occurs by the formation of imperfect “hairy ribbons” (1D) that interact laterally spontaneously to form a layer (2D) that interact in parallel to form mesoscale crystals (3D).

The new theoretical model developed by our research group,^[^ ^25^ ^]^ performs an energetic and topological analysis of the cluster and approximations of supramolecular structures, through the concept of compact packing in the crystal structure. Thus, a crystallization mechanism is proposed that seeks to highlight which interactions guide the approximation between chains (1D) for the formation of layers (2D), but also to determine at which stage the formation of the structure (3D) becomes more favorable than the growth of the layer (2D). In addition, the new model verifies the possibility of new supramolecular growth in a direction already observed, before the growth in the three directions. Therefore, the objective of this work is to elaborate proposals of crystallization mechanisms at the molecular level of halogenated benzyl alcohols using our theoretical model in the X‐ray data of these compounds.

## 2 Results and Discussion

### 2.1 Molecular Section

**Figure 1:**
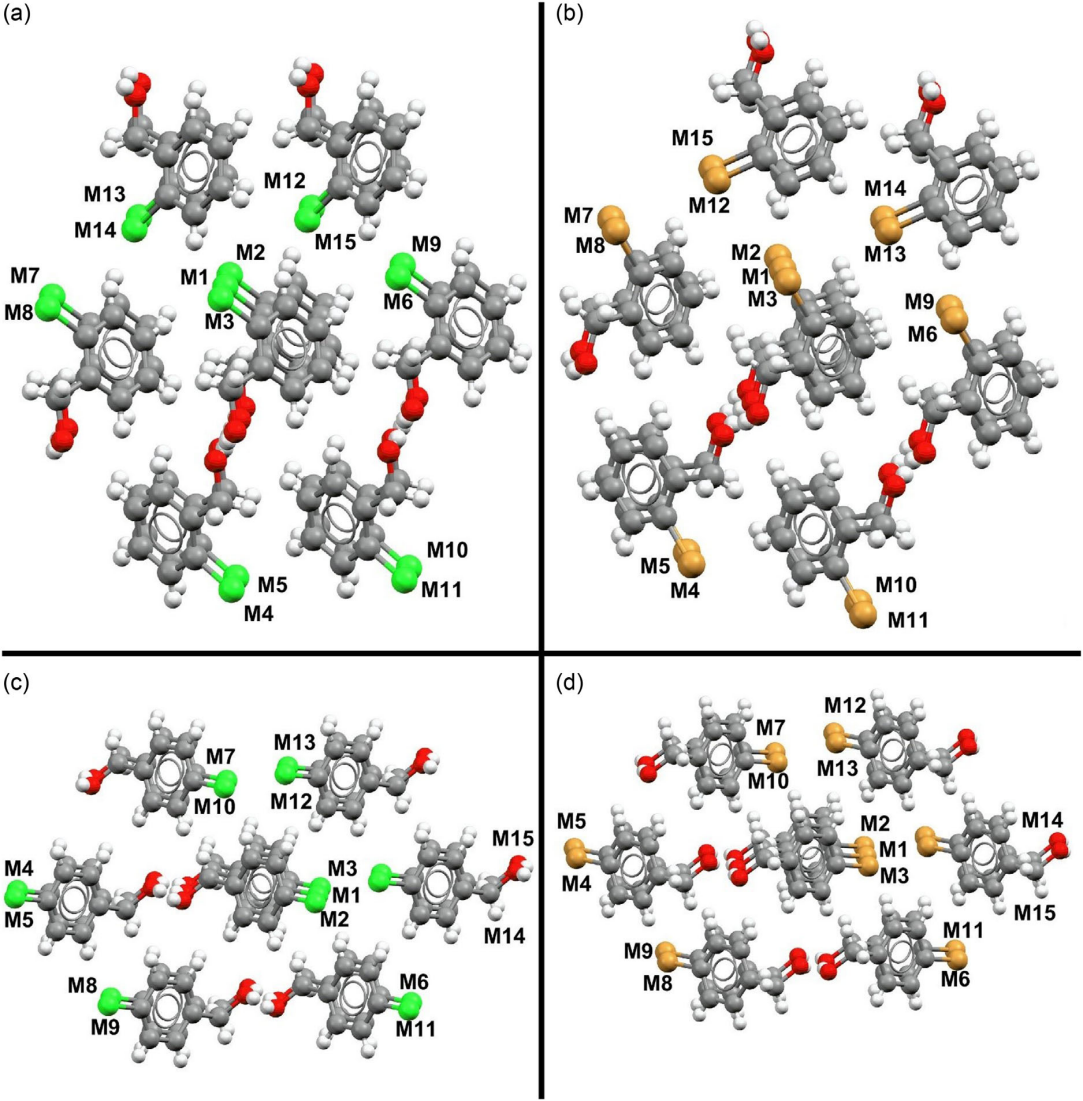
First sphere of coordination a) *o*‐chlorobenzyl alcohol, b) *o*‐bromobenzyl alcohol, c) *p*‐chlorobenzyl alcohol, and d) *p*‐bromobenzyl alcohol.

**Figure 2:**
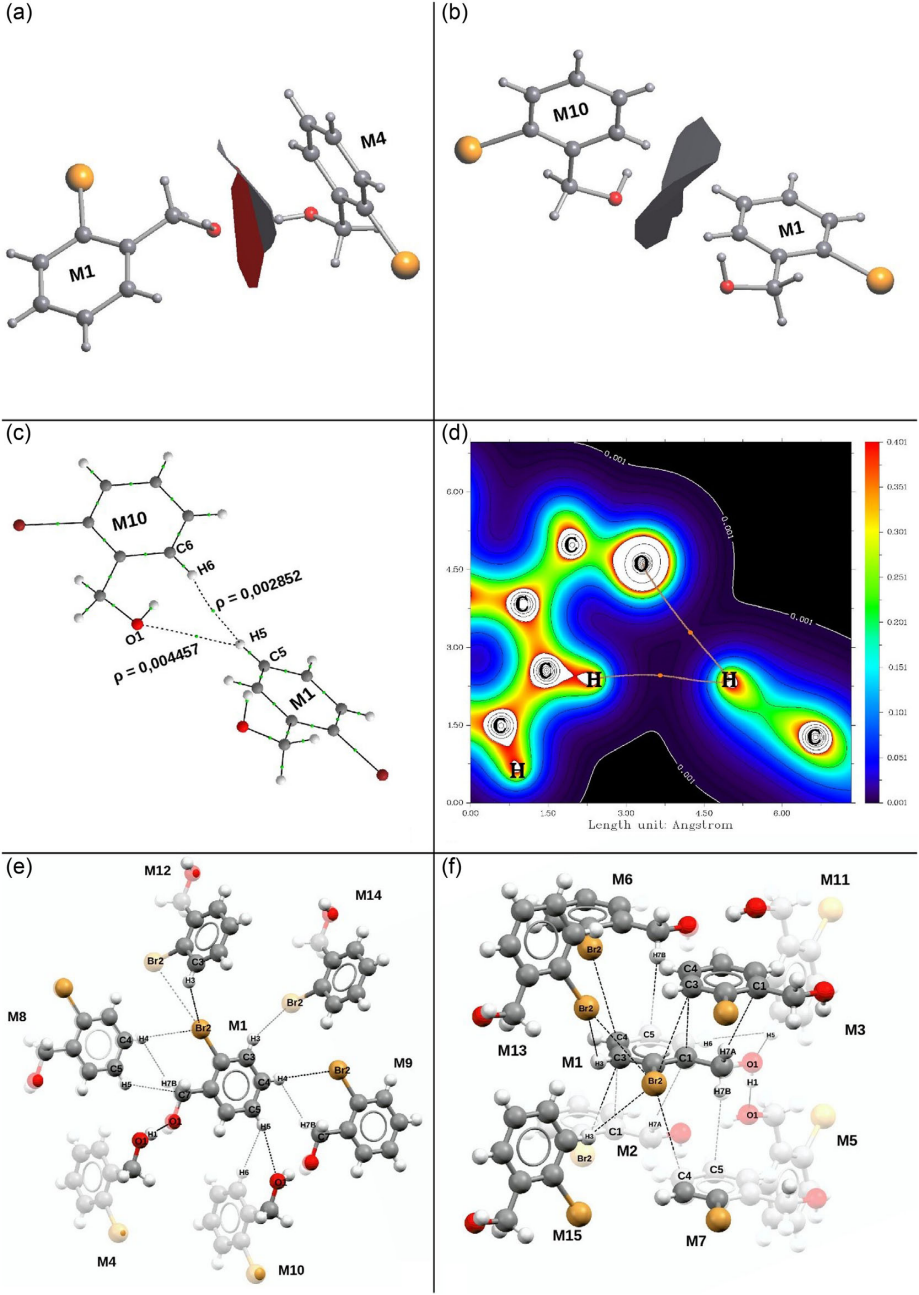
Contact between atoms: a,b) contact surface between M1 and peripheral molecules; c) representative molecules to exemplify the QTAIM analysis; d) QTAIM analysis; e) representation of all contacts of all molecules in the main plane with the central molecule (M1); and f) representation of the contacts of all molecules in the upper/lower plane with the central molecule (M1).

The class of compounds used for the study were halogenated benzyl alcohols. These molecules present a wide variety of interaction sites. Among them, the most notable is in the functional group itself (OH), in which the formation of classic hydrogen bonds (O—H···O) with oxygen is possible, acting as a proton donor or acceptor. The benzene ring tends to form strong π···π interactions and weak hydrogen bonds (C—H···H—C), with C—H···π interactions also being possible. The halogen atoms allow the formation of interactions via σ‐hole (X···X) and can act as acceptors in weak hydrogen bonds (C—H···X). This variety of possible interactions, together with the variation of the halogen atom and/or its position in the aromatic ring, can lead to different crystal structures with distinct nucleation mechanisms and physical properties. From the crystallographic data obtained, the first coordination sphere of each compound was determined using the Voronoi–Dirichlet Polyhedron method (VDP).^[^ ^26^ ^]^ The cluster was also evaluated topologically from the Hirshfeld surface (Figure S1, Supporting Information). Like that, all compounds presented a molecular coordination number (MCN) equal to 14 (**Figure** **1**) and each pair of molecules (M1···Mn) surrounding the central molecule (M1) had their contact surface determined by the VDP method (**Figure** **2**a,b). The calculation of interaction energy of all molecules in the cluster was performed by DFT at the ωB97X‐D3 level of theory^[^ ^27^ ^]^ with def2‐tzvp base^[^ ^28^ ^]^ using the ORCA program.^[^ ^29^ ^]^ The basis set superposition error (BSSE) error^[^ ^30^ ^]^ was corrected by counterpoise correction (CP), generating interaction energies (GM1···Mn). To determine the contact between atoms of each pair of molecules, the quantum theory of atoms in molecules (QTAIM) model^[^ ^31^ ^]^ was used exemplified in Figure 2c,d. For better visualization of the contact points, the cluster was divided into two images (Figure 2e,f).

**Figure 3:**
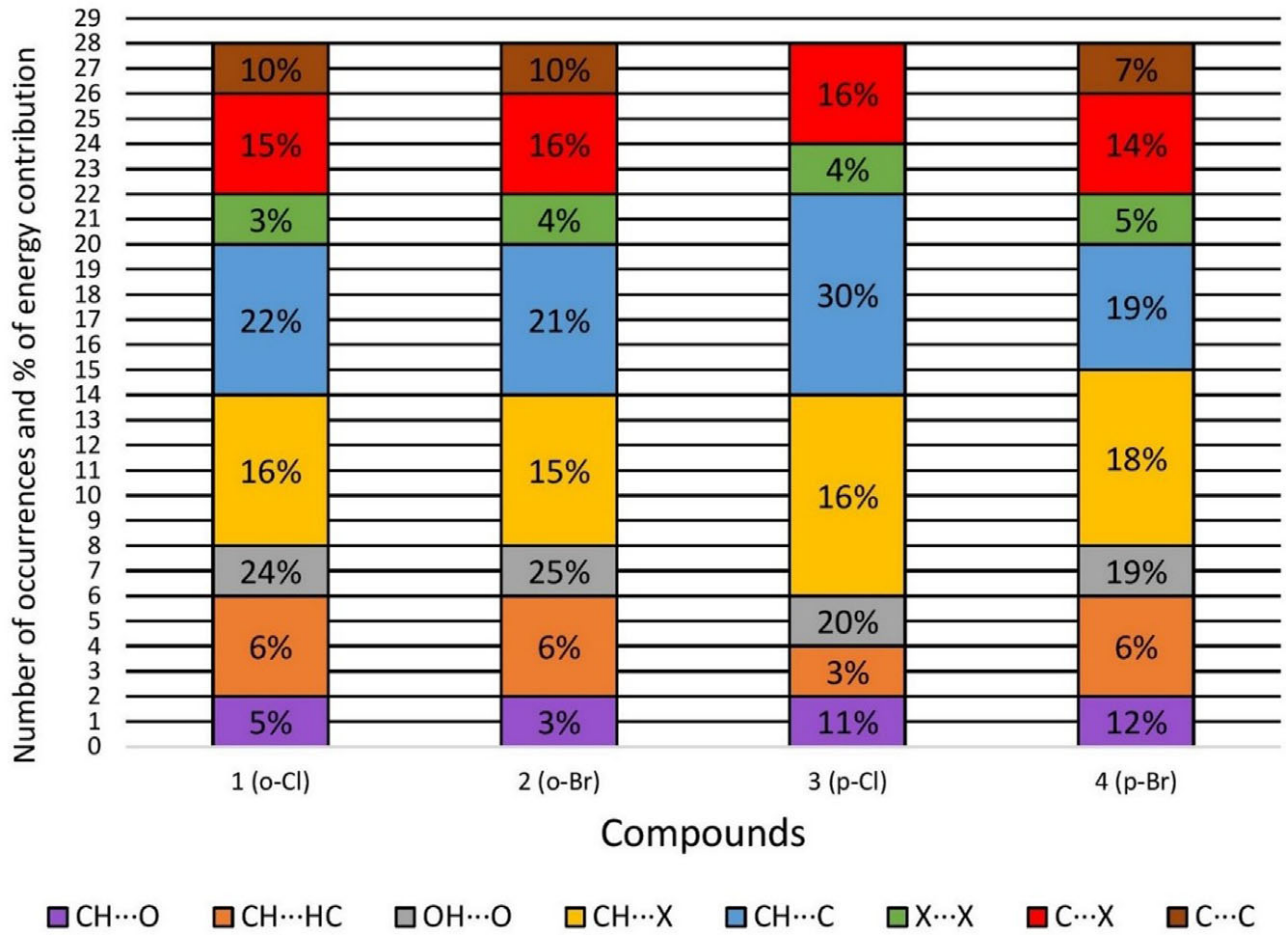
Number of occurrences and energetic contribution of each class of intermolecular interactions for each compound.

In *o*‐Br it is clear that the π stacking represented by the pairs M1‐M2 and M1‐M3 formed by the contact points Br···C, C···C, and C···H—C and with a contact area of 23.78 Å^2^ (**Table** **1**) are more stabilizing for the cluster than the classical OH···O (M1‐M4 and M1‐M5). All these contact area and interaction energy data for the other alcohols are available in Table S1–S3 and the interactions are represented in Figure S2–S4 of the Supporting Information. The robustness of these contacts was also evaluated globally through the number of occurrences and their energetic contribution to the stabilization of the first coordination sphere (Figure S5, Supporting Information) and for each compound (**Figure** **3**). In these compounds, it is notable that the CH···C, CH···X, and C···X interactions contribute with more than 50% (Figure S5, Supporting Information) to the stabilization of the analyzed clusters, while the O—H···O hydrogen bond contributes with only 22%. In order to investigate whether the interaction energy values found are appropriate, we compared these theoretical interaction energy data with experimental melting point data (Table S4, Supporting Information). Thereby, the correlation was notable so that the greater the lattice energy,^[^ ^32^ ^]^ the higher the melting point of the compounds analyzed (Table S4, Supporting Information). Detailed discussion of the supramolecular energetic and topological study of the first coordination sphere of halogenated benzyl alcohols is available in the Supporting information. The crystallization mechanism of halogenated benzyl alcohols was proposed based on our previously reported model.^[^ ^25^ ^]^

**Table 1:** M1···Mn molecule pair, contact area, interaction energy, interaction, interatomic distance, contact energy, contribution, and electron density for the supramolecular cluster of *o*‐bromo‐substituted benzyl alcohol.

Dimer	a)Contact area [Å^2^]	b)Interaction energy [kcal mol^−1^]	c)Interactions	Interatomic distance [Å]	d)Contact energy [kcal mol^−1^]	c)Contribution [%]	c) *ρ* (u.a.)
M1···M2	23.49	−6.57	C1···H7A–C7	2.983	−2.45	37	0.004746
			C3···Br2	3.831	−2.02	31	0.003929
			C4···C1	3.570	−2.10	32	0.004075
M1···M3	23.49	−6.57	Br2···C3	3.831	−2.02	31	0.00393
			C1···C4	3.569	−2.10	32	0.004075
			C7–H7A···C1	2.983	−2.45	37	0.004739
M1···M4	11.13	−5.48	O1···H1–O1	1.829	−5.48	100	0.031597
M1···M5	11.13	−5.48	O1–H1···O1	1.829	−5.48	100	0.031523
M1···M6	13.90	−2.79	C4···Br2	3.821	−1.37	49	0.004145
			C5···H7B–C7	2.951	−1.42	51	0.00431
M1···M7	13.90	−2.79	Br2···C4	3.822	−1.37	49	0.004145
			C7–H7B···C5	2.951	−1.42	51	0.00431
M1···M8	15.92	−2.12	Br2···H4–C4	3.138	−1.00	47	0.004855
			H7B···H4–C4	2.768	−0.52	25	0.00259
			C7···H5–C5	3.067	−0.60	28	0.002909
M1···M9	15.92	−2.12	C4–H4···Br2	3.137	−1.00	47	0.004857
			C4–H4···H7B–C7	2.768	−0.52	25	0.002592
			C5–H5···C7	3.066	−0.60	28	0.002902
M1···M10	12.06	−1.50	C5–H5···H6–C6	2.608	−0.59	39	0.002852
			C5–H5···O1	2.798	−0.91	61	0.004457
M1···M11	12.06	−1.50	O1···H5–C5	2.798	−0.59	39	0.002854
			C6–H6···H5–C5	2.608	−0.91	61	0.004462
M1···M12	7.76	−1.55	Br2···H3–C3	3.280	−0.64	41	0.003958
			Br2···Br2	3.780	−0.91	59	0.005663
M1···M13	7.76	−1.55	Br2···Br2	3.780	−0.91	59	0.005655
			C3–H3···Br2	3.281	−0.64	41	0.003959
M1···M14	12.46	−1.74	C3–H3···Br2	3.104	−1.74	100	0.005389
M1···M15	12.46	−1.74	Br2···H3–C3	3.104	−1.74	100	0.005389

a) ToposPro.

b) (G_M1···Mn_ = E_M1···Mn_ – 2*E_M1_) corrected by BSSE.

c) QTAIM (MultiWFN).

d) Contact energy = G_M1···Mn_*Contribution percentage.

This model uses crystallographic data obtained by X‐ray diffraction, combined with computational tools, to energetically and topologically analyze the interactions between molecules and supramolecular structures, such as chains and layers. With these data, it was possible to propose a crystallization mechanism based on the hierarchy of the most stabilizing intermolecular interactions, since the crystallization process is thermodynamically favored. Currently, several studies on self‐assembly in the crystallization process are in agreement with the nonclassical theory. For example, the study by Biran et al.^[^ ^23^ ^]^ states that the initial nucleation of perylene diimide occurs through stronger intermolecular interaction, resulting in the formation of supramolecular chains, these chains subsequently interact with each other to form a 2D structure. In a recent study by Elizabeth et al.^[^ ^24^ ^]^ it is observed that the crystallization of coronene dipeptide occurs initially by the formation of 1D “hairy ribbons” that act as a building block for the formation of 2D “flat ribbons”, which in turn merge to form increasingly larger 3D “ribbons”. Thus, the first step of the mechanism involves the strongest intermolecular interaction present in the crystal packing. In subsequent steps, the model proposes to determine and analyze the energy of supramolecular interactions that occur between chains (1D) and layers (2D). The formation of these structures occurs through the most stabilizing interaction. The crystallization mechanism is considered completed only when the 3D structure is formed and growth occurs in all directions of the unit cell, and has all interactions present in the first coordination sphere.

Although, the solvent plays an important role in crystallization, especially during the initial stages, our model^[^ ^25^ ^]^ does not require its inclusion in the calculations. This is because the methodology is based on the final crystal packing obtained by X‐ray diffraction. From this structure, we apply a retrocrystallization approach, energetically and topologically analyzing the intermolecular interactions present in the solid state. Therefore, the proposed mechanism considers only the stabilizing interactions responsible for crystal formation, without the need to simulate the solvent environment.

In this work, the stabilization energy of the intermolecular interactions between each pair of molecules selected within the first coordination sphere was calculated. This value was determined based on the difference between the interaction energy of a Mn molecule with an M1 molecule (E_M1···Mn_) and twice the energy of an isolated M1 molecule (E_M1_), as expressed by the equation G_M1···Mn_ = E_M1···Mn_ – 2E_M1_.^[^ ^14^ ^]^ This energy corresponds to the first stage of the crystallization mechanism. For the subsequent stages, the same principle is applied, but considering the interaction between two identical supramolecular structures (S_Mc_ and S_Mn_), according to the equation G_SMc···SMn_ = E_SMc···SMn_ – 2E_SMc_. When expansion of the previous stage is possible, the stabilization energy is calculated based on the interaction between a central supramolecular structure and two structures from the previous stage, using the equation G_SMc···2SMn_ = E_SMc···2SMn_ – E_SMc_ – 2E_SMn_, as previously described in our model.^[^ ^25^ ^]^

### 2.2 Supramolecular Section

The supramolecular structure is built from the application of the proposed model^[^ ^25^ ^]^ to suggest the crystallization mechanisms of halogenated benzyl alcohols that begins with obtaining crystallographic data by X‐ray diffraction. Based on this data, the first coordination sphere of each compound is determined using the VDP method, which defines the intermolecular contact surfaces. Based on the model, an energetic analysis of the interactions present in this coordination sphere is carried out, using calculations based on DFT. This analysis makes it possible to identify the most stabilizing intermolecular interaction, responsible for initiating the crystallization process, characterized by the formation of 1D chains by approximation between isolated molecules. Next, the model energetically evaluates the interactions obtained from approximation hypotheses between these supramolecular chains, which promote 2D growth and the formation of supramolecular layers. Finally, different approximation hypotheses between the layers are analyzed and the most stabilizing one is determined, which leads to the construction of the 3D supramolecular structure (Figure S6a, Supporting Information). The hypotheses are defined based on the contact surface between the supramolecular structures. In addition, the approximation of dimeric structures, the expansion of the previous step and growth along an already observed direction lead to an increase in the number of steps in the proposed crystallization mechanism (Figure S6b, Supporting Information).

By applying our model, stage 1 of the proposed crystallization mechanism for the compound *o*‐Br begins through a π stacking in parallel displacement with the C—H···π and Br···π contact points from the approach between two molecules (M1···M2 and M1···M3). This intermolecular interaction presents a contact area of 23.49 Å^2^, which corresponds to a stabilization energy of −6.57 kcal mol^−1^ (Table 1), considered the strongest interaction present in the first coordination sphere. Since this interaction can occur on both sides of the central molecule, it should lead to the formation of a chain of at least three molecules, which grows along the *a* axis. This chain presented a total energy of −13.30 kcal mol^−1^ referring to the intermolecular interactions. The value found is more stabilizing than twice that of just one interaction, since there are interactions between the peripheral molecules, as already observed in halogenated anilines.^[^ ^25^ ^]^

**Figure 4:**
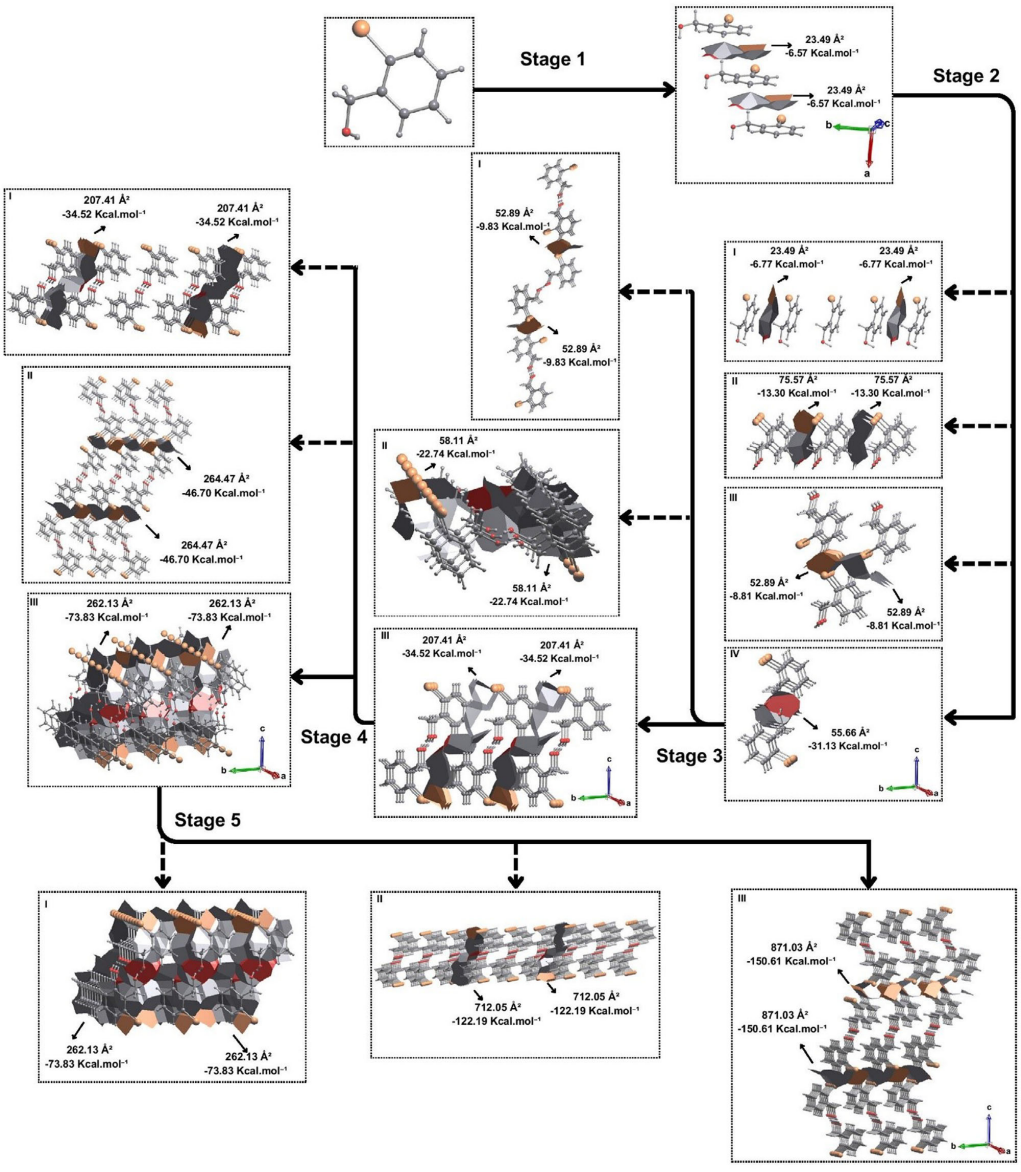
Proposal for the crystallization mechanism of *ortho*‐bromo‐substituted benzyl alcohol. For better understanding, view Video S1, Supporting Information.

In stage 2 of this mechanism, four hypotheses must be analyzed according to the largest contact areas between the chains. Hypothesis I evaluate the expansion of the previous step based on the same interactions, with the approximation of one molecule on each side of the chain (**Figure** **4**). Each interaction presented a stabilizing energy of −6.77 kcal mol^−1^. The stabilizing energy to be considered should be −13.54 kcal mol^−1^ (double) for the purpose of competition between the hypotheses (due to the need to have a similar number of molecules in a “fair” competition for stability). The hypotheses II, III, and IV were evaluated by approximating chains formed in the previous step in different orientations. Hypothesis II has a contact area of 75.57 Å^2^ between chains, with C—H···Br and C—H···H—C interactions, which has a stabilizing energy of −13.30 kcal mol^−1^. Hypothesis III has a contact area of 52.89 Å^2^, with C—H···Br and Br···Br interactions between chains, which has a stabilizing energy of −8.81 kcal mol^−1^. In addition, there is hypothesis IV with approximation between chains with a contact area of 55.66 Å^2^, smaller than hypothesis II, through an O···H—O contact that resulted in a higher stabilization energy of −31.13 kcal mol^−1^. With this, we can conclude that step 2 of the *o*‐Br crystallization mechanism must occur through hypothesis IV, with the approximation between two supramolecular chains in a dimeric form (Figure 4).

For stage 3, we analyzed three hypotheses for the crystallization mechanism of *o*‐Br. First, hypothesis I suggests C—H···Br and Br···Br interactions, in which a stabilization energy of −9.83 kcal mol^−1^ and a contact area of 52.89 Å^2^ were obtained. Hypothesis II reveals the approximation between chains by means of a π···π stacking, in which this approximation has a stabilization energy of −22.74 kcal mol^−1^ and a contact area of 58.11 Å^2^. For hypothesis III, a contact area of 207.41 (referring to the C—H···H—C, C—H···Br, and C—H···O contacts) and stabilization energies of −34.52 kcal mol^−1^ were found, indicating that step 3 of the proposed mechanism is guided by hypothesis III. That way, two structures approach a central structure along the *b* axis in both senses, forming a supramolecular layer (Figure 4).

For stage 4, three hypotheses must be investigated. Hypothesis I is precisely the continuation of the expansion of the previous step, from the same interactions, which has the same energy (−34.52 kcal mol^−1^) and growth along the same *b* axis. For the purpose of evaluating the competition, twice the energy of the supramolecular interaction (−69.04 kcal mol^−1^) must be considered. Hypotheses II and III of step 4 investigate the approximation between layers formed in step 3. In hypothesis II, this approximation has a contact area of 264.47 Å^2^ and a stabilization energy of −46.70 kcal mol^−1^ resulting from the supramolecular interactions C—H···Br and Br···Br, suggesting growth along the *c* axis. Finally, hypothesis III has a contact area of 262.13 Å^2^ and a stabilization energy of −73.83 kcal mol^−1^, indicating that step 4 of the proposed mechanism is guided by this hypothesis, generating a new π stacking along the *a* axis. Thus, two layers approach a central layer along the *a* axis in both senses (Figure 4). This hypothesis presented a smaller area and a higher stabilization energy when compared to hypothesis II.

So far, the C—H···Br and Br···Br interactions present in the first coordination sphere were not observed in the proposed mechanism. Thus, the need for a fifth stage was seen, in which three hypotheses were analyzed. The first hypothesis follows the expansion of the previous step with an energy of −73.83 kcal mol^−1^. Hypotheses II and III investigate the approximations of structures formed in the previous step that have stabilization energies of −122.19 and −150.61 kcal mol^−1^, respectively. These results indicate that step 5 should be guided by hypothesis III through the C—H···Br and Br···Br interactions, generating a growth along the *c* axis (Figure 4 and Video S1, Supporting Information), finalizing the proposed *o*‐Br mechanism.

**Figure 5:**
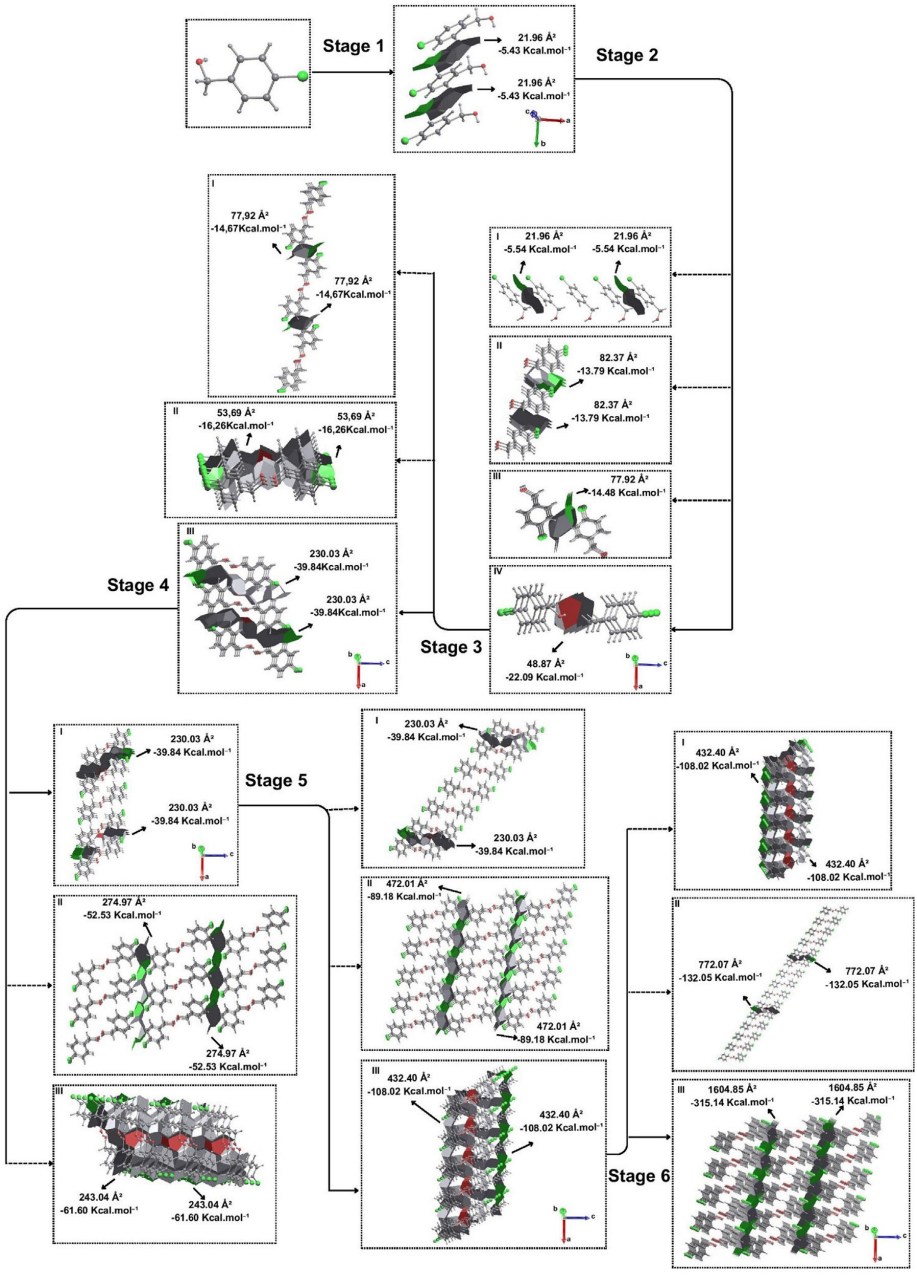
Proposal for the crystallization mechanism of *para*‐chloro‐substituted benzyl alcohol. For better understanding, view Video S2, Supporting Information.

For the compound *p*‐Cl, stage 1 of the proposed crystallization mechanism begins through a π stacking with the contact points C—H···π and Cl···π from the approximation between two molecules (M1···M2 and M1···M3). This intermolecular interaction presents a contact area of 21.96 Å^2^, which corresponds to a stabilization energy of −5.43 kcal mol^−1^, considered the strongest interaction present in the first coordination sphere. Since this interaction can occur on both sides of the central molecule, it should lead to the formation of a chain of at least three molecules, which grows along the *b* axis (**Figure** **5**). This chain presented a total energy of −10.96 kcal mol^−1^ referring to the intermolecular interactions, greater than twice that of an interaction mentioned due to the presence of interactions between peripheral molecules.

For stage 2 of this mechanism, four hypotheses were analyzed according to the largest contact areas between the chains. Hypothesis I evaluate the expansion of the previous stage from the same interactions, with the approach of one molecule on each side of the chain (Figure 5). Each interaction presented a stabilizing energy of −5.54 kcal mol^−1^. The stabilizing energy to be considered should be −11.08 kcal mol^−1^ (double) for the purpose of competition between the hypotheses. In hypothesis II, a contact area of 82.37 Å^2^ between chains was observed, with C—H···Cl and C—H···H—C interactions, and a stabilizing energy of −13.79 kcal mol^−1^. Hypothesis III has a contact area of 77.92 Å^2^, with C—H···Cl, C—H···H—C interactions between chains, which has a stabilizing energy of −14.48 kcal mol^−1^, differing from hypothesis II by one more contact through C—H···Cl interactions forming a supramolecular dimeric structure. Finally, hypothesis IV has an approximation between chains with a contact area of 48.87 Å^2^ (smaller than hypotheses II and III), through an O···H—O contact that resulted in a higher stabilization energy of −22.09 kcal mol^−1^. Thus, we can conclude that step 2 of the *p*‐Cl crystallization mechanism must occur through hypothesis IV, with the dimeric approximation (Figure 5) between two chains formed in the previous step.

For stage 3, three hypotheses were analyzed for the crystallization mechanism of *p*‐Cl. Initially, hypothesis I points to the C—H···Cl and C—H···C interactions with −14.67 kcal mol^−1^ of stabilization energy and 77.92 Å^2^ of contact area. Hypothesis II suggests the approximation between chains by means of a π···π stacking, in which this approximation has a stabilization energy of −16.26 kcal mol^−1^ and a contact area of 53.69 Å^2^. For hypothesis III, a contact area of 230.03 Å^2^ (referring to the C—H···H—C, C—H···Cl, C—H···O contacts) and interaction energies of −39.84 kcal mol^−1^ were found, indicating that step 3 of the proposed mechanism is guided by hypothesis III. Thus, two chains approach a central chain along the *a* axis in both senses, forming a supramolecular layer (Figure 5).

For stage 4, three hypotheses must be investigated. Hypothesis I will be the expansion of the previous step, which has the same energy (−39.84 kcal mol^−1^) and growth along the same *a* axis, and for the purpose of competition −79.68 kcal mol^−1^ (double) will be used. In hypothesis II, the approximation has a contact area of 274.97 Å^2^ and a stabilization energy of −52.53 kcal mol^−1^ resulting from the supramolecular interactions C—H···Cl and Cl···Cl, suggesting growth along the *c* axis. Finally, hypothesis III has a contact area of 243.04 Å^2^ and a stabilization energy of −61.60 kcal mol^−1^ through π···π interactions. When evaluating the three hypotheses, step 4 will be guided by the expansion of the previous step by increasing the supramolecular chain already formed (Figure 5), which demonstrated greater stabilization energy when compared to hypotheses II and III. Thus, the need for a fifth step was verified in order to verify the formation of a 3D structure and observe all the intermolecular interactions present in the first coordination sphere.

For stage 5, three new growth hypotheses were analyzed. For hypothesis I, a new expansion of the previous step should be considered, in which an energy of −79.68 kcal mol^−1^ was used for competition purposes. Hypothesis II will be the approximation of layers formed in the previous step by C—H···Cl and Cl···Cl interactions that demonstrated a contact area of 472.01 Å^2^ and a stabilization energy of −89.18 kcal mol^−1^. Hypothesis III points to π···π interactions, which present a contact area of 432.40 Å^2^ and an energy of −108.02 kcal mol^−1^, resulting in a higher stabilization energy, which generates an approximation between layers along the *b* axis (Figure 5). However, the need for a new step is perceived, since the previous growth pointed along the *b* axis, which had already been observed in step 1. Again, due to the lack of H···Cl and Cl···Cl type interactions demonstrated in the first coordination sphere concomitant with the lack of growth along the *c* axis, it was necessary to add a sixth step.

In stage 6, three hypotheses were again analyzed. Hypothesis I refer to the expansion of the previous stage, demonstrating an energy of −216.04 kcal mol^−1^ for competition purposes. Hypothesis II analyzes the C—H···H—C and C—H···Cl interactions, which generated a contact area of 772.07 Å^2^ and a stabilization energy of −132.05 kcal mol^−1^. Finally, hypothesis III points to Cl···Cl and C—H···Cl interactions, in which a contact area of 1604.85 Å^2^ and an energy of −315.14 kcal mol^−1^ were found. This value is significantly higher than the other hypotheses of this step, which should guide step 6 along the *c* axis (Figure 5 and Video S2, Supporting Information). It reveals all the intermolecular interactions present in the first coordination sphere and thus finalizes the crystallization mechanism of the *para*‐chloro‐substituted benzyl alcohol.

**Figure 6:**
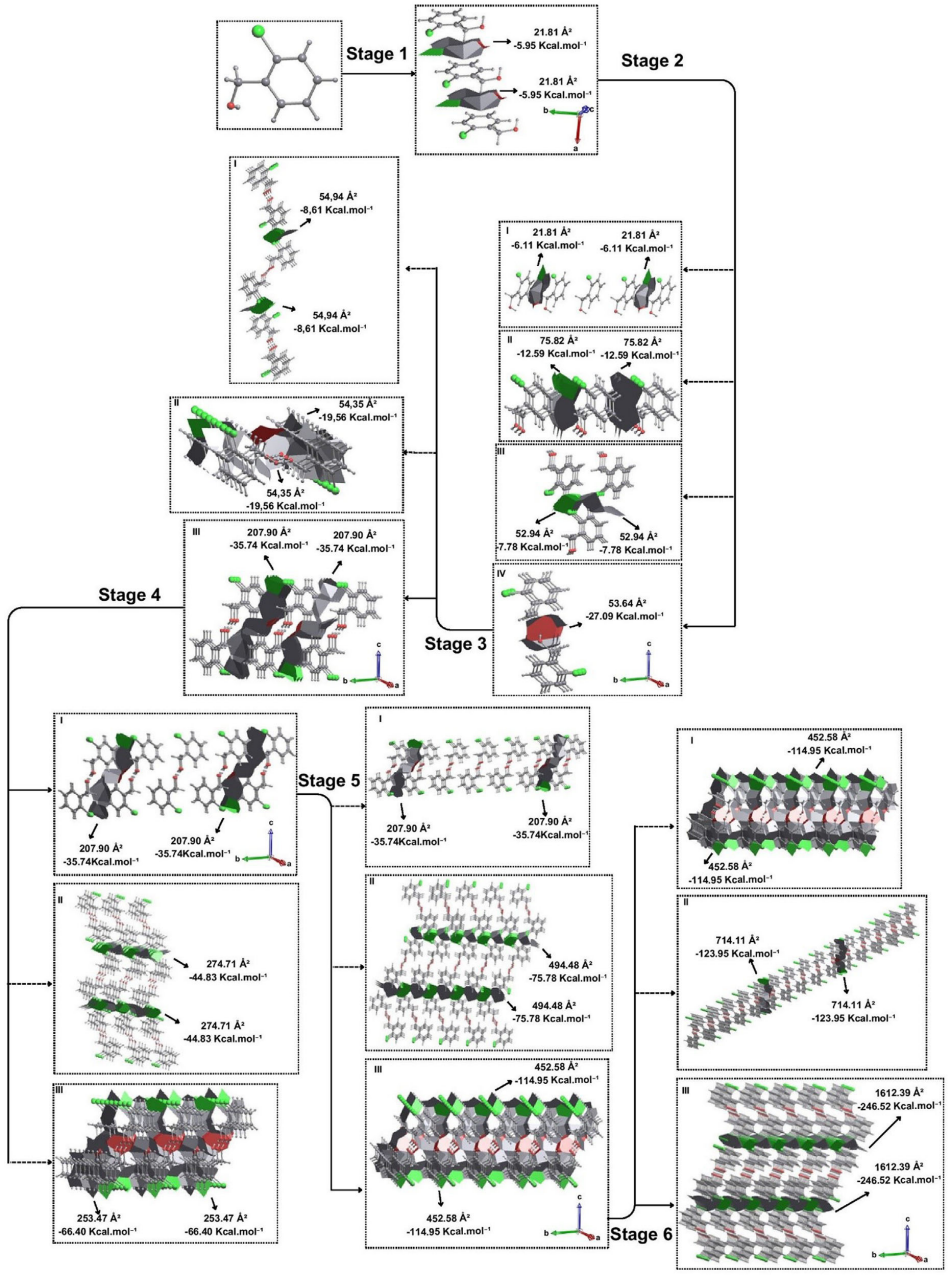
Proposal for the crystallization mechanism of *ortho*‐chloro‐substituted alcohol. For better understanding, view Video S3, Supporting Information.

For the compounds substituted in the *ortho* position, the phenomenon of isostructuralism was observed. Similarly, the same was observed for the *para*‐substituted compounds, which are isostructural to each other. Similar hypotheses were observed for both compounds. Therefore, only the thermodynamically favored hypotheses will be described for these cases. However, the description and representation of the proposed crystallization mechanism for *para*‐bromo benzyl alcohol will be approached in the Supporting information (Figure S7, Supporting Information). In stage 4, the *o*‐Cl presents a peculiar characteristic in his proposed crystallization mechanism, different from its isostructural *o*‐Br. In step 1 of *o*‐Cl nucleation, the approximation of C—H···π and Cl···π interactions was observed with a contact area of 21.81 Å^2^ and energy of −5.95 kcal mol^−1^, leading to the formation of a chain (**Figure** **6**) of at least three molecules due to the approximation of both sides of the central molecule by the *a* axis. In step 2, we will have the formation of a supramolecular dimer (Figure 6) between chains through an O···H—O contact with 53.64 Å^2^ of contact area and interaction energy of −27.09 kcal mol^−1^. Step 3 occurs through C—H···H—C, C—H···Cl, and C—H···O interactions with 207.90 Å^2^ of contact area and interaction energy of −35.74 kcal mol^−1^, forming a supramolecular layer (Figure 6) along the *b* axis. For the next step, it was observed that the hypotheses, despite being similar to *o*‐Br, had different energetic behavior. Therefore, for step 4, three hypotheses of interactions analogous to *o*‐Br were evidenced. Initially, hypothesis I is the expansion of the previous step considering an energy of −71.48 kcal mol^−1^ for competition purposes. In hypothesis II this approximation has a contact area of 274.71 Å^2^ and stabilization energy of −44.83 kcal mol^−1^ resulting from the supramolecular interactions C—H···Cl and Cl···Cl, suggesting growth along the *c* axis. Hypothesis III has a contact area of 253.47 Å^2^ and a stabilization energy of −66.40 kcal mol^−1^. This indicates that this step, unlike *o*‐Br, will have its step 4 guided by the expansion of the interactions of the previous step (Figure 6). It was therefore necessary to analyze a fifth stage in order to highlight all the intermolecular interactions present in the first coordination sphere concomitant with the formation of a 3D structure. For step 5 again, hypothesis I was investigated the expansion from the C—H···H—C, C—H···Cl, and C—H···O interactions with the same area and energy for competition purposes. For hypothesis II with C—H···Cl and Cl···Cl interactions, a contact area of 494.48 Å^2^ and an energy of −75.78 kcal mol^−1^ were observed. Hypothesis III occurs through the same π stacking (C—H···π and Cl···π) and the same growth axis already evidenced in stage 1 of nucleation, in which they form an area of 452.58 Å^2^ and energy of −114.95 kcal mol^−1^. Thus, step 5 of the *o*‐Cl crystallization mechanism occurs through hypothesis III with growth along the *a* axis (Figure 6). Again, in order to evidence the end of crystallization by C—H···Cl and Cl···Cl interactions and growth along the *c* axis, a sixth crystallization step was followed. For step 6 of the crystallization mechanism, three hypotheses were analyzed, the first hypothesis being guided by the expansion of the previous step, with the same area but with an energy of −229.9 kcal mol^−1^ for competition purposes. Hypothesis II investigates approximations through C—H···H—C, C—H···Cl and C—H···O interactions, in which an energy of −123.95 kcal mol^−1^ and a contact area of 714.11 Å^2^ are observed. Finally, hypothesis III is given by C—H···Cl and Cl···Cl interactions with an area of 1612.39 Å^2^ and an energy of −246.52 kcal mol^−1^, indicating growth along the *c* axis. It can be concluded that hypothesis III guides stage 6 of the proposed crystallization mechanism for the *o*‐Cl (Figure 6 and Video S3, Supporting Information).

Benzyl alcohols initiated their mechanism from π‐stacking, instead of classical hydrogen bonds, similar to what has already been observed in most halogenated anilines.^[^ ^25^ ^]^ Unlike benzoic acids^[^ ^21^ ^]^ and halogenated benzamides^[^ ^20^ ^]^ that initiated their mechanisms by strong classical hydrogen bonds, probably due to the formation of supramolecular dimers. Analogous to other classes of halogenated compounds, benzyl alcohols have the completion of their proposed crystallization mechanisms from C—H···X and X···X interactions, as well as benzoic acids,^[^ ^21^ ^]^ benzamides,^[^ ^20^ ^]^ phenols,^[^ ^19^ ^]^ and anilines.^[^ ^25^ ^]^

In order to understand the factors that influenced the nucleation stages presented in the *o*‐Br and *o*‐Cl compounds, which are isostructural to each other, in which the *o*‐Br compound has a crystallization proposal in 5 steps and the *o*‐Cl in 6 steps, an energy study of the atomic contacts in step 4 of both was carried out. It is important to note that steps 3 and 4 of the *o*‐Cl compound are presented separately for didactic reasons, although their interactions and growth along the same axis are identical. In practice, we can consider them as a single step.^[^ ^25^ ^]^

**Figure 7:**
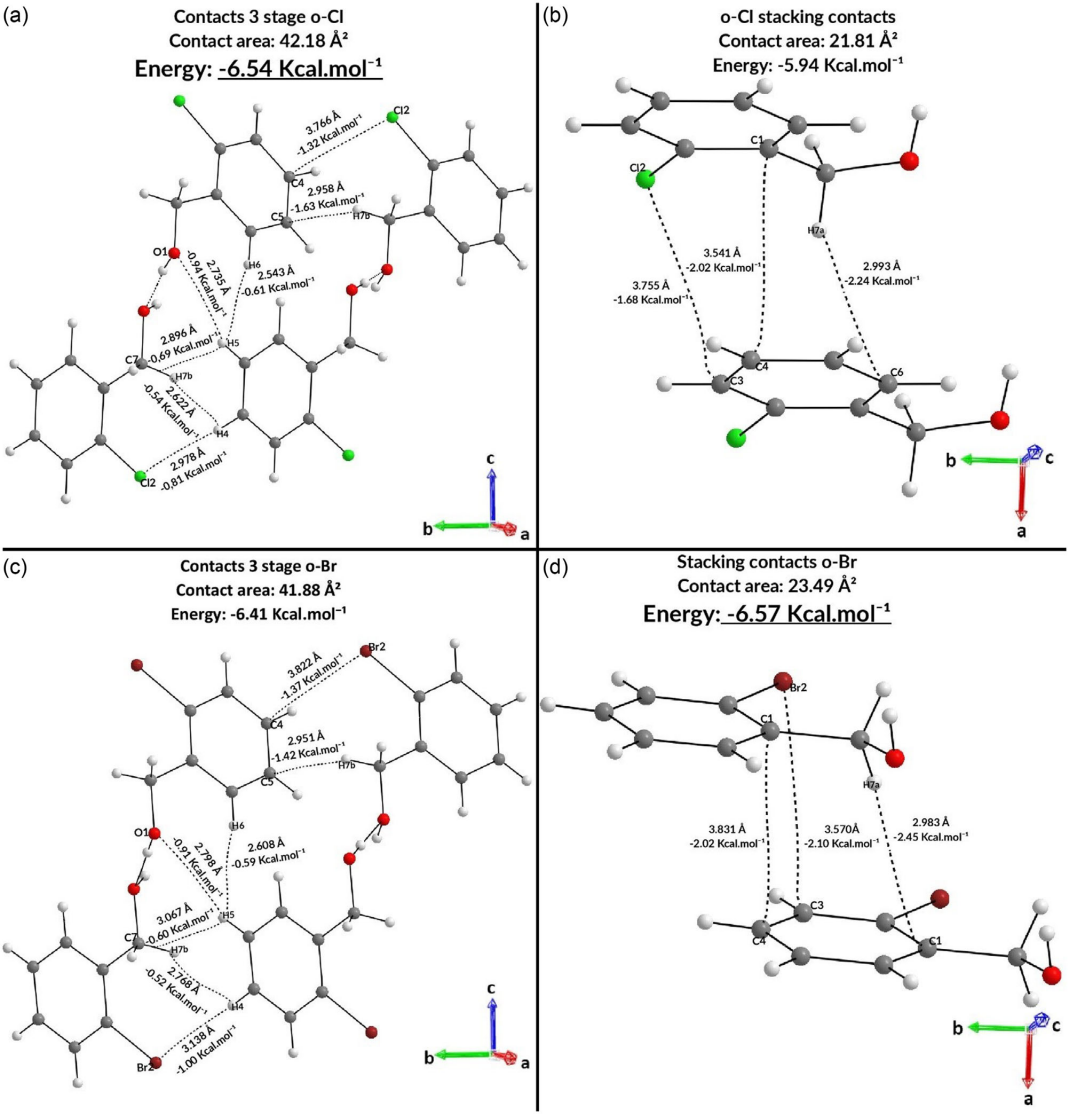
Comparison between hypotheses of substituted *o*‐chloro and *o*‐bromo alcohols.

Thus, we considered it necessary to carry out an analysis linked to the halogen variation and how they influenced the crystallization stages through the intermolecular interactions present in step 4 of both compounds, this being the different step between the compounds. For the *o*‐Br compound, step 4 is due to hypothesis III by new growth by π‐stacking along the *a* axis. For the *o*‐Cl compound, step 4 is given by hypothesis I guided by the expansion of the previous step along the *b* axis. Therefore, it is possible to state that in step 4 there is fierce competition between hypotheses I and III in the two benzyl alcohols. Thus, hypothesis I is guided by interactions X···C, C—H···C, X···H—C, C—H···H—C, and O···H—C and hypothesis III by C—H···π, X···π, and π···π in both *ortho*‐substituted compounds. Based on the observed interactions, it is possible to relate the interactions already evidenced in the unit cell, therefore, hypothesis I refers to the interactions of the pairs M1···M2/M1···M3 and hypothesis III to the pairs M1···M6/M1···M7, M1···M8/M1···M9, and M1···M10/M1···M11 in both the *o*‐Cl and *o*‐Br compounds. With the interactions that define the steps already established (**Figure** **7**), it is possible to analyze the sum of the energy of the interactions that guide hypothesis I of step 4 of both compounds. This results in a value of −6.41 kcal mol^−^^1^ for *o*‐Br and −6.54 kcal mol^−^^1^ for *o*‐Cl.

When the same analysis is performed for the interactions that guide hypothesis III of step 4, the values of −6.57 and −5.95 kcal mol^−1^ are obtained for *o*‐Br and *o*‐Cl, respectively. When comparing the sum of the interactions of the first coordination sphere that guide hypothesis I of step 4, the energy of −6.41 kcal mol^−1^ obtained for *o*‐Br is not enough to surpass the value of −6.57 kcal mol^−1^ of hypothesis III; however, for *o*‐Cl, −6.54 kcal mol^−1^ easily surpasses −5.95 kcal mol^−1^. This fact can be explained by three parameters: contact area, interatomic distance, and the electron density of the intermolecular interactions involved. It is worth noting that the analysis of the unit cell alone is often not sufficient to describe the crystallization mechanism. In this case, it was only possible to use it because both hypothesis I of each compound and hypothesis III of each compound have the same number of contact points through the same interactions, both verified by the QTAIM analysis. Based on the data linked to the first coordination sphere, it is believed that *ortho*‐substituted compounds have a different energy behavior during the crystallization stages. This is due to the size of the halogen, which can directly influence the contact areas and interatomic distances. Larger halogens allow for a more stabilizing π‐stacking due to greater polarizability (Figure 7d). Bettens et al.^[^ ^33^ ^]^ state that less bulky halogens favor peripheral intermolecular interactions with smaller interatomic distances (Figure 7a), favoring them energetically.^[^ ^34^ ^]^ When comparing hypotheses I and III of step 4, by means of their contact points (Figure 7), the influence of both factors related to the size of the halogens can be seen: π‐stacking is favored in the *o*‐Br alcohol, while peripheral interactions are favored in the *o*‐Cl alcohol. The pairs M1···M6/M1···M7 show that the C4···X2 interactions are less stabilizing for the *o*‐Cl compound, but the C5···H7B‐C7 interactions are more stabilizing for it because they have a smaller interatomic distance due to the smaller space occupied by the halogen.

The same phenomenon occurs in the M1···M8/M1···M9 pairs, in which the X2···H4‐C4 interaction is less stabilizing (Figure 7) for the *p*‐chloro‐substituted compound (Table S3, Supporting Information), but the adjacent interactions (C7‐H7B···H4‐C4, C7···H5‐C5) are more stabilizing when compared to the *p*‐bromo‐substituted compound (Table 1). However, for the π stacking, represented by the pairs M1···M2/M1···M3 and the contacts C1···C4, X2···C3 and C6···H7A‐C7, although the interactions occur over greater distances for the *o*‐Br compound, all the contacts shown are more stabilizing (Table 1—Figure 7). This is possibly due to the polarization of the bromine (greater van der Waals radius), which allows a more stabilizing approximation with the aromatic ring. In hypothesis I, the contacts in step 4 of *o*‐Cl are more stabilizing, with a smaller interatomic distance, greater electron density and larger contact area. In the π stacking interactions (hypothesis III), the *o*‐Br compound presents a larger contact area and a greater stabilizing energy. Analyzing the *para*‐substituted compounds it is possible to show that in relation to the position of the halogen the *para*‐substituted compounds stabilize the dimers M1···M6/M1···M7, M1···M8/M1···M9, and M1···M10/M1···M11 more than the *ortho*‐substituted standard.^[^ ^33^ ^]^ Therefore, for these compounds, the sum of these interactions easily exceeds the π stacking energy, thus always generating an expansion in step 4.

At the end of the elaboration of the proposals for crystallization mechanisms of halogenated benzyl alcohols, a correlation was noted between the growth axes of the unit cell and the morphology of the crystals, since the crystalline habit resembles the unit cell.^[^ ^35^ ^]^ In the four compounds studied, it was found that the crystallization process began in the direction of the smallest axis of the unit cell and ended along its largest axis, similar to that observed in halogenated anilines.^[^ ^25^ ^]^

## 3 Conclusion

In this study, we used the model developed by our research group to propose crystallization mechanisms at the molecular level. This model is mainly based on theoretical data on the energy and topology of intermolecular and supramolecular interactions, which in this work was applied to halogenated benzyl alcohols in the *ortho* or *para* positions. All compounds presented a MCN equal to 14. Weaker interactions, such as C—H···X, C—H···C, and C···X, played a fundamental role in stabilizing the crystal packing, due to the larger contact area of these fragments and their greater occurrence, when compared to stronger interactions such as O—H···O. As observed in halogenated anilines, the model showed that it is crucial to calculate the energy resulting from supramolecular interactions between the contact surfaces of supramolecular chains or layers also in halogenated alcohols. In this work, it was observed that weaker interactions, when cooperative, can prevail over stronger interactions in the search for crystal stability. Even so, all compounds studied had their steps guided by the hierarchy of energies of intermolecular interactions evidenced in the first coordination sphere. When applying the model, it was observed that a supramolecular structure can grow along an axis more than once, in an intercalated manner, before all three axes have their growth observed, a factor that was evidenced in all compounds analyzed. This characteristic is evidenced in step 4 of *o*‐Br and in steps 5 of compounds *o*‐Cl, *p*‐Cl, and *p*‐Br, where the reappearance of π stacking between supramolecular structures is observed. This factor energetically favored the appearance of more contact points of halogen bonds X···X due to the π stacking generated previously, thus allowing growth along the last axis of the unit cell for all compounds. Another feature described in the model that increased the number of steps was the approximation of dimeric supramolecular structures via O—H···O hydrogen bonds, which was observed in the second step of all studied compounds. Our model allows us to thermodynamically assess whether a structure will continue to grow in one direction and when growth should occur in a second direction. The expansion characteristic, which is observed when a structure grows continuously along the same axis, was evidenced in step 4 of the compounds *o*‐Cl, *p*‐Cl, and *p*‐Br, with the exception of the alcohol *o*‐Br. From a topological and energetic analysis of the compounds, it is possible to conclude that bromine, when compared to chlorine in the *ortho* position, allows a more stabilizing π stacking. Therefore, in step 4, where there would be expansion of the previous step, π stacking was favored. It was also possible to note that the position of the halogen in the ring directly influenced the steps of the crystallization mechanism, being able to increase or decrease the number of steps, as well as, generating structures with distinct supramolecular architectures. However, when exclusively analyzing the halogen variation, it was observed that it only added an expansion step, maintaining growth along the same axes in *ortho*‐chloro compared to its isostructural (*o*‐Br), without causing significant variation in the supramolecular architecture. Thus, the analysis of intermolecular and supramolecular interactions, in topological and energetic terms, is a crucial tool for understanding the crystallization process at the molecular level. This will help researchers to prepare organic supramolecular structures in the solid state with specific properties in the development of crystal engineering.

## 4 Experimental Section

The single‐crystal X‐ray diffraction crystallographic data was obtained from the Cambridge Structural Database (CSD), using ConQuest Version 2022.3.0.^[^ ^36^ ^]^ The compounds studied have the following CCDC/CSD deposition numbers: 2003971 for *o*‐Cl, 882251 for *o*‐Br, 192470 for *p*‐Cl, and 192472 for *p*‐Br. Initially, the MCN^[^ ^37^ ^]^ of each compound and the contact surface (M1···Mn) between the molecules in the cluster were determined by the VDP,^[^ ^38^ ^]^ using the ToposPro^[^ ^39^ ^]^ program. Distances involving hydrogen atoms from X‐ray diffraction data were automatically corrected by the Crystal Explorer.^[^ ^40^ ^]^ The Hirshfeld surfaces were determined using the Crystal Explorer^[^ ^40^ ^]^ program. In this work, the stabilization energy of the intermolecular interactions of each pair of molecules selected in the first coordination sphere was calculated. This value was obtained from the difference between the energy of a Mn molecule interacting with the M1 molecule (E_M1···Mn_) and the energy of twice an isolated M1 molecule (E_M1_) using the equation G_M1···Mn_ = E_M1···Mn_ – 2E_M1_.^[^ ^14^ ^]^ This energy was used to describe the first stage of the crystallization mechanism. For the other stages, the same method was used; however, the S_Mc_···S_Mn_ interaction occurs between two equal supramolecular structures, as expressed in the equation G_SMc···SMn_ = E_SMc···SMn_ – 2E_SMc_. When we had the possibility of expanding the previous stage, the stabilizing energy was determined by the S_Mc_···2S_Mn_ interaction between a central supramolecular structure and two supramolecular structures from the previous stage, as shown in the equation G_SMc···2SMn_ = E_SMc···2SMn_ – E_SMc_ – 2E_SMn_. The lattice energy was determined by the sum of the stabilizing energy of the intermolecular interactions of the M1···Mn dimers of the first coordination sphere; in the presence of identical dimers (same interatomic distance, contact area, interaction energy, and electronic density), the energy of only one of these dimers is considered for the calculation, since it is given per mol. This energy was then correlated with the melting point.^[^ ^32^ ^]^ These energies were determined by single point gas phase calculations (no structural optimization) at the ωB97X‐D3^[^ ^27^ ^]^ level of theory, using the def2‐TZVP^[^ ^28^ ^]^ basis set and the RIJCOSX^[^ ^41^ ^]^ approximation with the auxiliary bases def2/J^[^ ^42^ ^]^ and def2‐TZVP/C^[^ ^43^ ^]^ in the ORCA (Version 5.0.3)^[^ ^29^ ^]^ program. All compounds used the default PModel initial guess method from the version of ORCA 5.0.3. The BSSE was calculated using Boys and CP^[^ ^30^ ^]^ method. The interactions involved, as well as their electronic densities, were determined by ORCA 5.0.3, MultiWFN (Version 3.7)^[^ ^44^ ^]^ and AIMAII (Version 19.10.12).^[^ ^45^ ^]^ The electronic density of the interactions was used to calculate the percentage contribution of each interaction using the following equation, contribution (%) = *ρ*_contact_/Σ*ρ*_interaction_ which is converted into the contact energy = G_M1···Mn_*contribution (%). The inputs for the energy calculations were made in the Avogadro (Version 1.2.0)^[^ ^46^ ^]^ program. The figures were made using ToposPro,^[^ ^39^ ^]^ Crystal Explorer,^[^ ^40^ ^]^ and Mercury (Version 2022.3.0).^[^ ^47^ ^]^

## Conflict of Interest

The authors declare no conflict of interest.

## Supporting information

Supplementary Material

## Data Availability

The data that support the findings of this study are available in the supplementary material of this article.
