# Supplementary material for: Proposal of Molecular‐Level Crystallization Mechanism for Halogenated Benzyl Alcohols: A Study of Isostructural Crystals
Source: Chempluschem. 2025 Jun 20;90(8):e202500144. doi: 10.1002/cplu.202500144 (PMC12352734; doi:10.1002/cplu.202500144)
Supplement: Supplementary file 1 — Supplementary Material [file CPLU-90-e202500144-s001.zip › cplu202500144-sup-0002-SuppData-S2.pdf]

ChemPlusChem

Supporting Information

PROPOSAL OF MOLECULAR-LEVEL CRYSTALLIZATION MECHANISM FOR HALOGENATED BENZYL ALCOHOLS: A STUDY OF ISOSTRUCTURAL CRYSTALS

Patrick Teixeira Campos[a]*, Pedro Henrique Cunha do Couto[a], Álex Canez Lemos Souza[a], Juliano Alex Roehrs[a]

[a] Laboratório de Química Orgânica Sintética, Estrutural e Computacional (LaQuiOSEC)
Instituto Federal de Educação, Ciência e Tecnologia Sul-rio-grandense (IFSul) – Câmpus Pelotas
CEP 96015-360, Pelotas, Brazil
E-mail: patrickcampos@ifsul.edu.br

Table of Content

Figure S1. Figure S1. Hirshfeld surface (a) Central molecule M1 with Hirshfeld surface surrounded by six molecules in the same plane, (b) six molecules with Hirshfeld surface surrounding the central molecule; (c) molecules with Hirshfeld surface in the plane including M1; (d) Hirshfeld surface of the molecules in the upper and lower planes including M1. 3

Figure S2. Supramolecular cluster showing central molecule with neighboring molecules (a) in the same plane and (b) in the upper and lower layers for o-Chloro-substituted benzyl alcohol. 4

Figure S3. Supramolecular cluster showing central molecule with neighboring molecules (a) in the same plane and (b) in the upper and lower layers for p-Bromo-substituted benzyl alcohol. 4

Figure S4. Supramolecular cluster showing central molecule with neighboring molecules (a) in the same plane and (b) in the upper and lower layers for p-Chloro-substituted benzyl alcohol. 5

Table S1. M1···Mn molecule pair, contact area, interaction energy, interaction, interatomic distance, contact energy, % contribution and electron density for the supramolecular cluster of p-Bromo-substituted benzyl alcohol. 6

Table S2. M1···Mn molecule pair, contact area, interaction energy, interaction, interatomic distance, contact energy, % contribution and electron density for the supramolecular cluster of p-Chloro-substituted benzyl alcohol. 7

Table S3. M1···Mn molecule pair, contact area, interaction energy, interaction, interatomic distance, contact energy, % contribution and electron density for the supramolecular cluster of o-Chloro-substituted benzyl alcohol. 8

Figure S5. Number of occurrences and energetic contribution of each class of intermolecular interactions for all compounds. 9

Table S4. Lattice energy and melting point of halogenated benzyl alcohols. 10

Figure S6. Generic demonstration of the application of the model a) Conventional crystallization route; b) Factors influencing the number of steps. 11

Figure S7. Proposed crystallization mechanism for p-Bromo substituted benzyl alcohol. 13

References 14

Based on data obtained from the CSD database,[36] the first coordination sphere of each compound was determined using the Voronoi–Dirichlet Polyhedron (VDP)[38] method. The importance of this structure lies in the fact that it is the smallest representative portion of the crystal in terms of intermolecular interactions. Thus, the cluster formed by the first coordination sphere encompasses all the intermolecular interactions present in the single crystal.

The Molecular Coordination Numbers (MCN)[37] were determined, indicating the number of molecules (Mn) around a central molecule (M1). All compounds analyzed presented an MCN of 14, with the most recurrent configuration in the literature for crystalline organic structures consisting of 6 molecules arranged in a ring around M1, 4 above and 4 below M1. These patterns are illustrated in Figure S1, where the analysis of the Hirshfeld surfaces for the two regions of the first coordination sphere of o-Br is presented. The topological evaluation highlights the importance of the contact surface between molecules in the construction of the first coordination sphere. This approach considers all molecules that have some contact area with M1, and not just those with interatomic distances equal to or less than the sum of the Van der Waals radii of the atoms involved in the interaction (red regions of Figure S1). In this context, the presence of a contact surface between molecules is treated as evidence of the existence of intermolecular interactions, while the interatomic distance is considered only as a criterion for comparing the strength of similar interactions.

After determining the supramolecular clusters for each compound and dividing these clusters into the main plane and upper/lower plane regions, the study moves on to calculate the energetic and topological parameters, which are essential to support the proposed crystallization mechanisms. This data makes it possible to evaluate intermolecular interactions and identify correlations between the structure and physical properties of the crystal. To identify the atoms involved in the interactions, we used the Quantum Theory of Atoms in Molecules (QTAIM) model,[31] which defines the contacts between pairs of molecules based on the molecular electronic density (ρ), a quantitative parameter calculated by DFT.

In the QTAIM model, contacts are identified by the presence of critical bond points (BCP) between atoms of different molecules. These critical points are defined as places where the gradient of the electronic density is zero, and the path of greatest variation in the electronic density describes the contact. The electronic density, being a value assigned to each point in space, is represented on a plane of interest, defined by the three atoms closest to the contact surface of the pair of molecules. In this way, the position of the analyzed point can be represented in two dimensions, with the electronic density displayed on a rainbow-like color scale, where values below the scale are represented in black and above in white. This analysis makes it possible to highlight the contacts responsible for intermolecular interaction and quantify their intensity, since the electronic density at the critical point is proportional to the stabilizing energy of the contact. With this approach, it is possible to gain a more in-depth understanding of the most relevant interactions for cluster stabilization.

The interactions between the molecules of the main plane and the central molecule M1 were analyzed, specifically in the case of o-Br, which has 6 pairs of molecules (Figure 2e). The M1···M10 interaction presented an energy of -1.50 $kcal.mol^{-1}$ and a contact area of 12.06 $Å^2$. The QTAIM analysis revealed two contacts in this interaction: C5-H5···H5-C5 and C5-H5···O1, with electron densities at the binding critical points of $2.852x10^{-3}$ and $4.457x10^{-3}$ a.u., respectively. Thus, it was possible to decompose the total interaction energy into the contributions of each contact, resulting in -0.91 and -0.59 $kcal.mol^{-1}$ for the respective contacts (Table 1). Interaction data for other compounds are presented in Tables S1–S3.

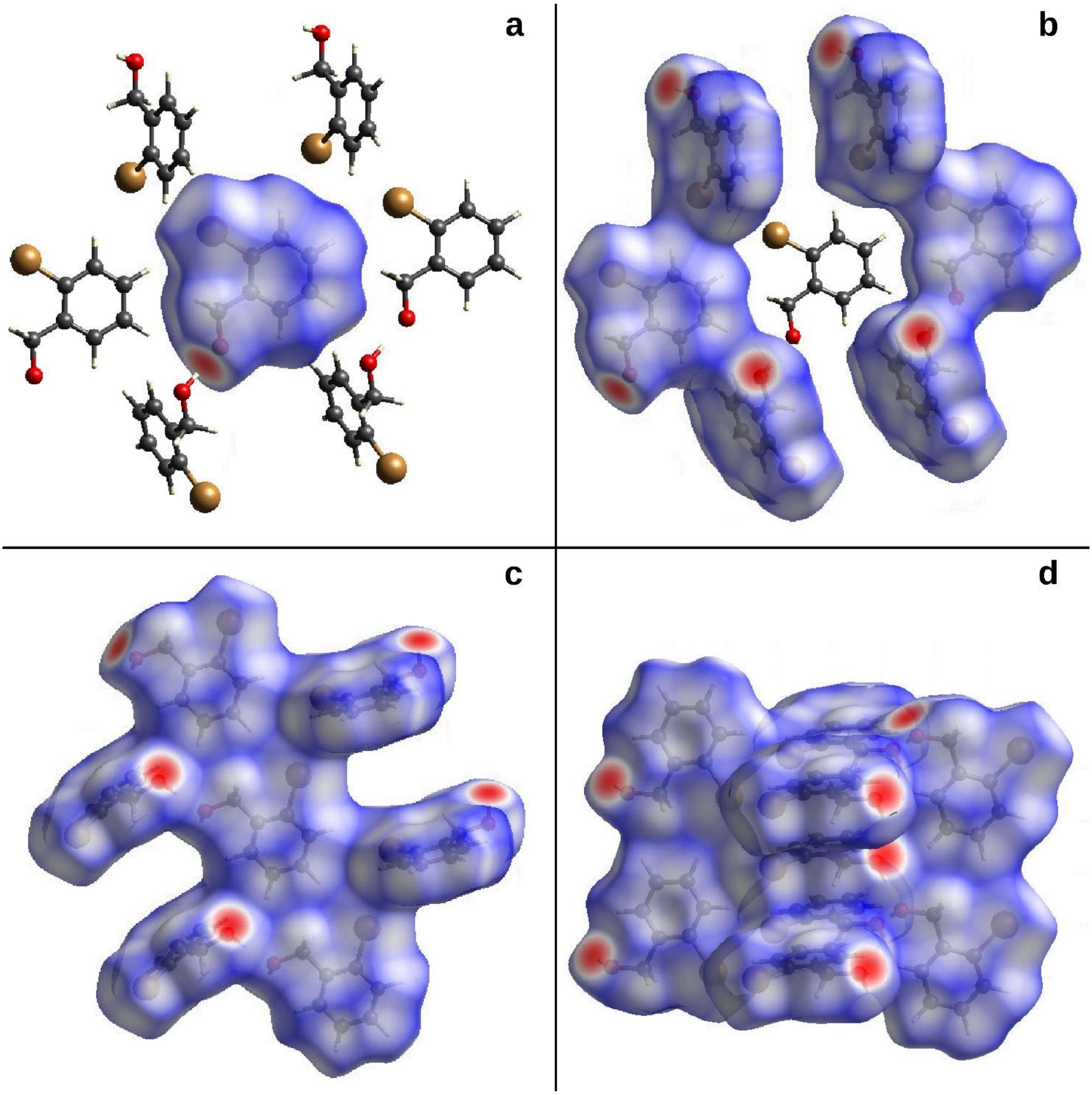

Figure S1. Figure S1. Hirshfeld surface (a) Central molecule M1 with Hirshfeld surface surrounded by six molecules in the same plane, (b) six molecules with Hirshfeld surface surrounding the central molecule; (c) molecules with Hirshfeld surface in the plane including M1; (d) Hirshfeld surface of the molecules in the upper and lower planes including M1.

The compounds studied showed a pattern of symmetry in the interactions of the first coordination sphere, so that M1···M11 is identical to M1···M10. The M1···M2 and M1···M3 interactions, for example, in the substituted o-Br alcohol exhibited energies of -6.57 kcal.mol^{-1} and a contact area of 23.49 Å^2 each. The large contact area between these molecules resulted in a greater number of contacts, including C-H···X, C-H···C and C···X. Other pairs of molecules were also evaluated, and the results are summarized in Table S1, both for the main plane molecules (Figure 2e) and for the upper and lower regions (Figure 2f). For the other compounds, their representations of the main plane and the upper and lower regions are illustrated in Figures S2-S4, while the corresponding pairs of molecules are presented in Tables S1-S3.

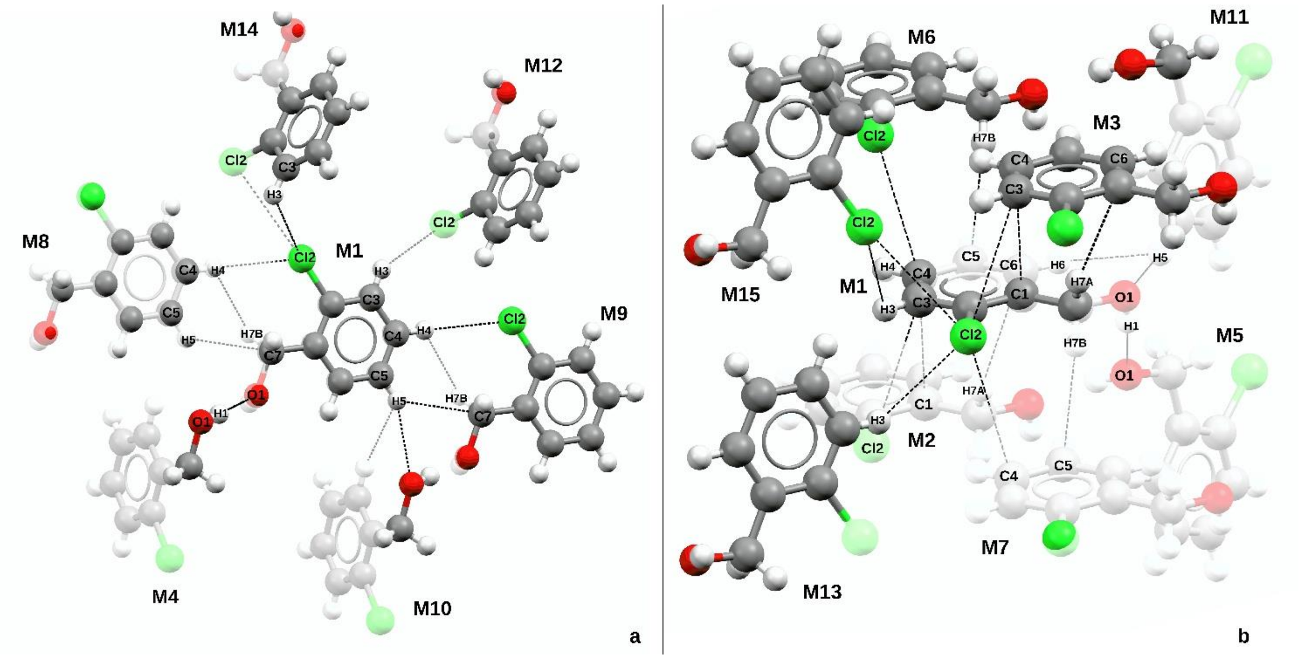

Figure S2. Supramolecular cluster showing central molecule with neighboring molecules (a) in the same plane and (b) in the upper and lower layers for o-Chloro-substituted benzyl alcohol.

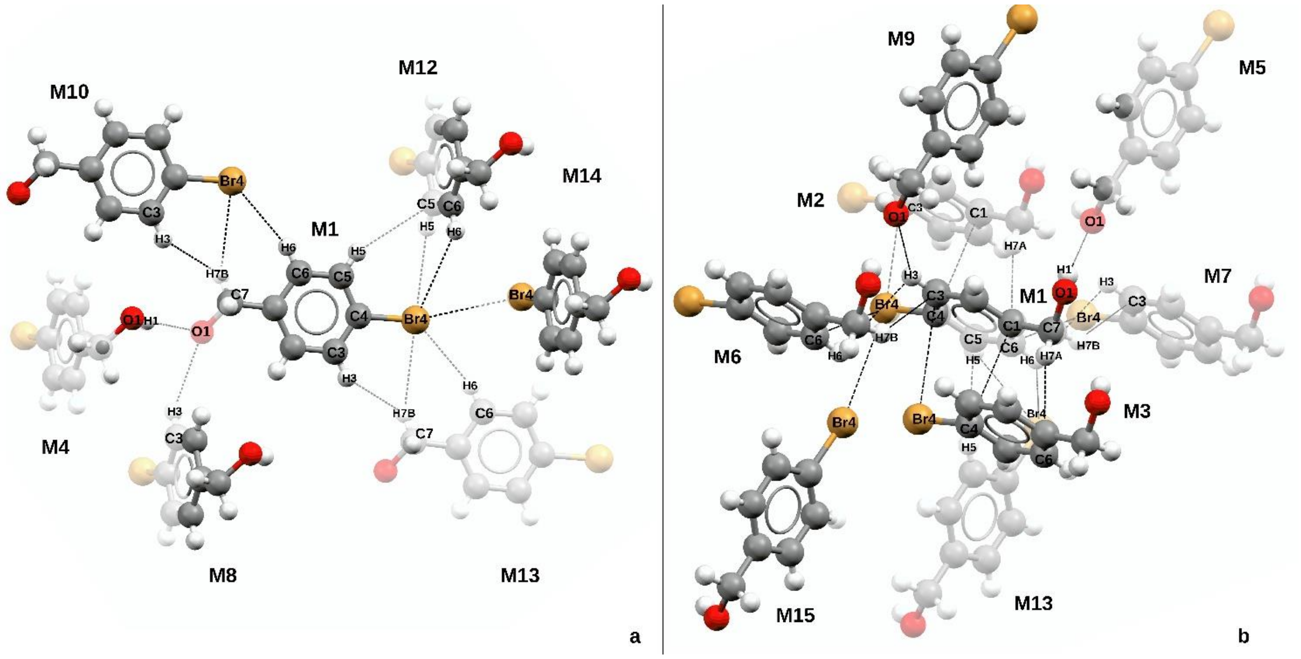

Figure S3. Supramolecular cluster showing central molecule with neighboring molecules (a) in the same plane and (b) in the upper and lower layers for p-Bromo-substituted benzyl alcohol.

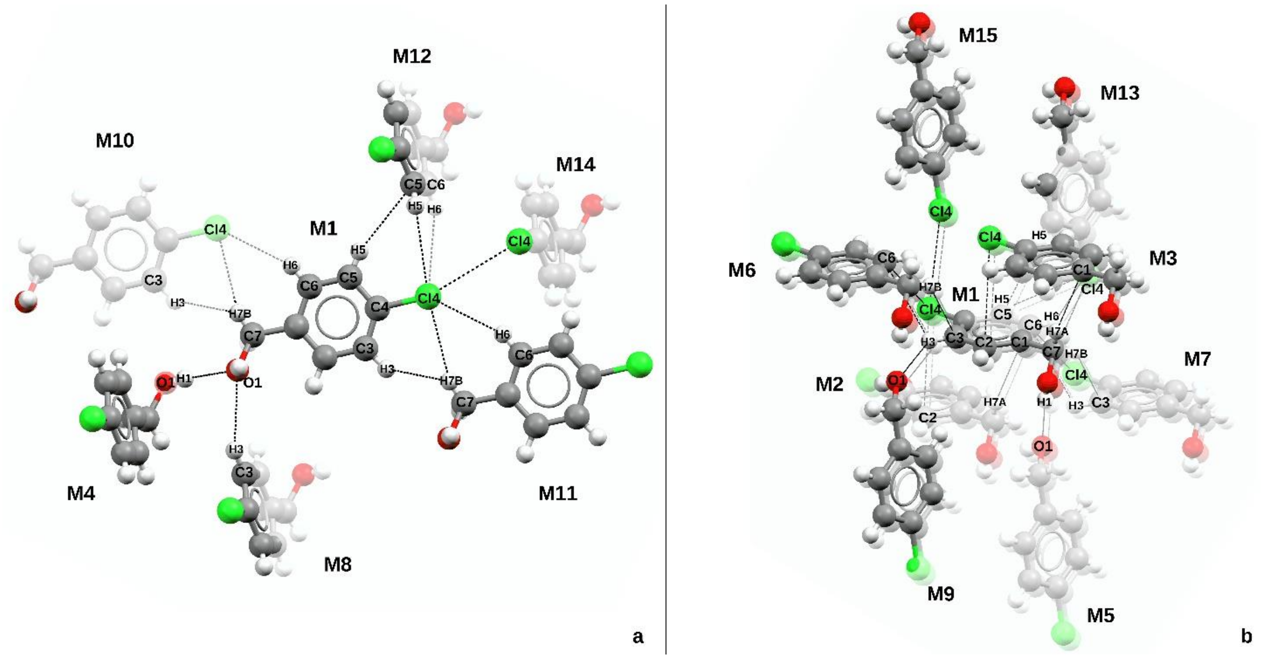

Figure S4. Supramolecular cluster showing central molecule with neighboring molecules (a) in the same plane and (b) in the upper and lower layers for p-Chloro-substituted benzyl alcohol.

Table S1. M1···Mn molecule pair, contact area, interaction energy, interaction, interatomic distance, contact energy, % contribution and electron density for the supramolecular cluster of p-Bromo-substituted benzyl alcohol.

Dimer	[a] Contact area (Å²)	[b]Interaction energy (kcal.mol⁻¹)	[c]Interações	Interatomic distance (Å)	[d] Contact energy (kcal.mol⁻¹)	[c] Contribution (%)	[c]ρ (u.a.)
M1···M2	23.78	-5.80	Br4···C3	3.882	-1.91	33	0.004233
			C4···C1	3.658	-1.57	27	0.003482
			C6···H7A-C7	2.945	-2.32	40	0.005143
M1···M3	23.78	-5.80	C7-H7A···C6	2.945	-2.32	40	0.005134
			C1···C4	3.658	-1.57	27	0.003482
			C3···Br4	3.882	-1.91	33	0.004237
M1···M4	9.68	-3.97	O1···H1-O1	2.144	-3.97	100	0.014868
M1···M5	9.68	-3.97	O1-H1···O1	2.144	-3.97	100	0.014872
M1···M6	17.78	-2.99	Br4···C6	4.053	-1.12	38	0.002774
			C3···H7B-C7	3.286	-0.97	32	0.002387
			C3-H3···H6-C6	3.103	-0.90	30	0.002217
M1···M7	17.78	-2.99	C7-H7B···C3	3.286	-0.97	32	0.002386
			C6-H6···H3-C3	3.103	-0.90	30	0.002217
			C6···Br4	4.052	-1.12	38	0.002776
M1···M8	13.63	-2.56	O1···H3-C3	2.741	-2.56	100	0.004895
M1···M9	13.63	-2.56	C3-H3···O1	2.741	-2.56	100	0.004896
M1···M10	14.26	-2.11	C6-H6···Br4	3.055	-0.86	41	0.005421
			C7-H7B···H3-C3	2.657	-0.43	20	0.002727
			C7-H7B···Br4	3.182	-0.82	39	0.005154
M1···M11	14.26	-2.11	C3-H3···H7B-C7	2.657	-0.43	20	0.002729
			Br4···H7B-C7	3.182	-0.82	39	0.005154
			Br4···H6-C6	3.055	-0.86	41	0.005422
M1···M12	16.07	-2.82	C5-H5···C5	3.151	-0.66	24	0.002778
			Br4···H5-C5	3.206	-1.15	40	0.004798
			Br4···H6-C6	3.286	-1.01	36	0.004207
M1···M13	16.07	-2.82	C6-H6···Br4	3.286	-1.01	36	0.004207
			C5-H5···Br4	3.205	-1.15	40	0.004799
			C5···H5-C5	3.150	-0.66	24	0.002777
M1···M14	4.42	-0.95	Br4···Br4	3.628	-0.95	100	0.007610
M1···M15	4.42	-0.95	Br4···Br4	3.628	-0.95	100	0.007610

a: ToposPro; **b:** ($G_{M1\cdots Mn} = E_{M1\cdots Mn} - 2*E_{M1}$) corrected by BSSE; **c:** QTAIM (MultiWFN);
d: Contact energy = $G_{M1\cdots Mn}$*Contribution percentage.

Table S2. M1···Mn molecule pair, contact area, interaction energy, interaction, interatomic distance, contact energy, % contribution and electron density for the supramolecular cluster of p-Chloro-substituted benzyl alcohol.

Dimer	[a] Contact area (Å²)	[b]Interaction energy (kcal.mol^{-1})	[c]Interações	Interatomic distance (Å)	[d] Contact energy (kcal.mol^{-1})	[c] Contribution (%)	[c]ρ (u.a.)
M1···M2	21.96	-5.43	Cl4···C2	3.878	-2.23	41	0.003364
			C1···H7A-C7	2.993	-3.20	59	0.004815
M1···M3	21.96	-5.43	C2···Cl4	3.877	-2.23	41	0.003361
			C7-H7A···C1	2.993	-3.20	59	0.004817
M1···M4	9.77	-4.15	O1···H1-O1	2.006	-4.15	100	0.020602
M1···M5	9.77	-4.15	O1-H1···O1	2.006	-4.15	100	0.020604
M1···M6	18.15	-3.12	Cl4···C6	3.898	-1.07	34	0.002826
			C3-H3···C6	3.291	-1.09	35	0.002875
			C3···H7B-C7	3.271	-0.96	31	0.002551
M1···M7	18.15	-3.12	C6···H3-C3	3.290	-1.09	35	0.002874
			C6···Cl4	3.898	-1.07	34	0.002824
			C7-H7B···C3	3.272	-0.96	31	0.00255
M1···M8	13.06	-2.21	O1···H3-C3	2.774	-2.21	100	0.004655
M1···M9	13.06	-2.21	C3-H3···O1	2.774	-2.21	100	0.004656
M1···M10	13.96	-2.01	C6-H6···Cl4	3.021	-0.69	34	0.004421
			C7-H7B···Cl4	3.091	-0.72	36	0.004631
			C7-H7B···H3-C3	2.47	-0.60	30	0.003888
M1···M11	13.96	-2.01	Cl4···H6-C6	3.021	-0.69	34	0.004426
			Cl4···H7B-C7	3.091	-0.72	36	0.004635
			C3-H3···H7B-C7	2.47	-0.60	30	0.003888
M1···M12	15.58	-2.74	Cl4···H5-C5	3.158	-0.87	32	0.003713
			Cl4···H6-C6	3.241	-0.96	35	0.004092
			C5-H5···C5	3.163	-0.91	33	0.003881
M1···M13	15.58	-2.74	C5-H5···Cl4	3.241	-0.96	35	0.004095
			C6-H6···Cl4	3.158	-0.87	32	0.003717
			C5···H5-C5	2.977	-0.91	33	0.003881
M1···M14	4.12	-0.73	Cl4···Cl4	3.585	-0.73	100	0.005303
M1···M15	4.12	-0.73	Cl4···Cl4	3.585	-0.73	100	0.005297

a: TopoSPro; **b:** ($G_{M1\cdots Mn} = E_{M1\cdots Mn} - 2*E_{M1}$) corrected by BSSE; **c:** QTAIM (MultiWFN);
d: Contact energy = $G_{M1\cdots Mn}$*Contribution percentage.

Table S3. M1···Mn molecule pair, contact area, interaction energy, interaction, interatomic distance, contact energy, % contribution and electron density for the supramolecular cluster of o-Chloro-substituted benzyl alcohol.

Dimer	[a] Contact area (Å²)	[b]Interaction energy ($kcal.mol^{-1}$)	[c]Interações	Interatomic distance (Å)	[d] Contact energy ($kcal.mol^{-1}$)	[c] Contribution (%)	[c]ρ (u.a.)
M1···M2	21.81	-5.94	C3···Cl2	3.755	-1.68	28	0.003607
			C6···H7A-C7	2.993	-2.24	38	0.004807
			C4···C1	3.541	-2.02	34	0.004335
M1···M3	21.81	-5.94	Cl2···C3	3.755	-1.68	28	0.003606
			C7-H7A···C6	2.993	-2.24	38	0.004803
			C1···C4	3.541	-2.02	34	0.004335
M1···M4	10.73	-5.00	O1···H1-O1	1.932	-5.00	100	0.0244
M1···M5	10.73	-5.00	O1-H1···O1	1.932	-5.00	100	0.024401
M1···M6	13.35	-2.95	C4···Cl2	3.766	-1.32	45	0.003644
			C5···H7B-C7	2.958	-1.63	55	0.004476
M1···M7	13.35	-2.95	Cl2···C4	3.766	-1.32	45	0.003643
			C7-H7B···C5	2.959	-1.63	55	0.004472
M1···M8	16.37	-2.04	Cl2···H4-C4	2.978	-0.81	40	0.005108
			C7-H7B···H4-C4	2.622	-0.54	26	0.003431
			C7···H5-C5	2.896	-0.69	34	0.004368
M1···M9	16.37	-2.04	C4-H4···Cl2	2.978	-0.81	40	0.005109
			C4-H4···H7B-C7	2.622	-0.54	26	0.00343
			C5-H5···C7	2.896	-0.69	34	0.004368
M1···M10	12.46	-1.55	C5-H5···O1	2.735	-0.94	61	0.005089
			C5-H5···H6-C6	2.543	-0.61	39	0.00332
M1···M11	12.46	-1.55	O1···H5-C5	2.735	-0.94	61	0.00509
			C6-H6···H5-C5	2.543	-0.61	39	0.003317
M1···M12	13.54	-1.86	C3-H3···Cl2	2.954	-1.86	100	0.005718
M1···M13	13.54	-1.86	Cl2···H3-C3	2.954	-1.86	100	0.005718
M1···M14	7.16	-1.22	Cl2···Cl2	3.637	-0.65	54	0.004707
			Cl2···H3-C3	3.19	-0.57	46	0.004078
M1···M15	7.16	-1.22	Cl2···Cl2	3.637	-0.65	54	0.004714
			C3-H3···Cl2	3.190	-0.57	46	0.004078

a: ToposPro; **b:** ($G_{M1\cdots Mn} = E_{M1\cdots Mn} - 2*E_{M1}$) corrected by BSSE; **c:** QTAIM (MultiWFN);
d: Contact energy = $G_{M1\cdots Mn}$*Contribution percentage.

From the interaction energies calculated by QTAIM, obtained by decomposing the energies of the pairs of molecules in each of their contacts, we sought to comprehensively evaluate the strength of these interactions and their energetic contribution to stabilizing the first coordination sphere, both for each compound and globally. The data was organized in graphs that allow the identification of patterns in the classes of intermolecular interactions of the compounds studied.

Figure S5 shows the number of occurrences of each type of interaction and their respective percentage energy contribution to the stabilization of the clusters in the four compounds analyzed. Figure 3 in the article presents this data individually for each compound. Classical hydrogen bonds, represented by the O-H···O class, showed the highest average stabilizing energy recorded, with -4.65 $kcal.mol^{-1}$, contributing 22% to the stabilization of the clusters, although they were only observed 8 times in total. On the other hand, contacts from the C-H···C class, although they have a significantly lower average energy of -1.53 $kcal.mol^{-1}$, contributed 23% to stabilizing the clusters, thanks to their abundance, with 25 occurrences in all the compounds. Although they are considered weak interactions, C-H···C contacts contributed 30% to the stabilization of the first coordination sphere of p-Cl, while O-H···O interactions contributed 20% to the same compound. Two other relevant contacts in terms of occurrence were those involving halogens: C-H···X and C···X, with average stabilizing energies of -1.01 $kcal.mol^{-1}$ and -1.59 $kcal.mol^{-1}$, respectively. These contacts were frequent and together contributed 31% of the total energy, playing a significant role in stabilizing the clusters. The C-H···X contact was recorded 27 times in all the compounds, with an energetic contribution of between 15% and 18% to the stabilization of the first coordination sphere (Figure 3), while the C···X contact, with 16 occurrences, showed a contribution of between 14% and 16%, values close to those of the C-H···X contact. In addition to these main classes, four other types of contact showed less energetic importance, but are still relevant to crystal packing. Contacts such as C-H···O and C-H···H-C contributed 6% and 5% respectively to the energy of the clusters. The lowest total energy contributions were 4% for σ-hole halogen bonds, represented by the X···X class, and 7% for the π···π interaction between aromatic rings, represented by the C···C class.

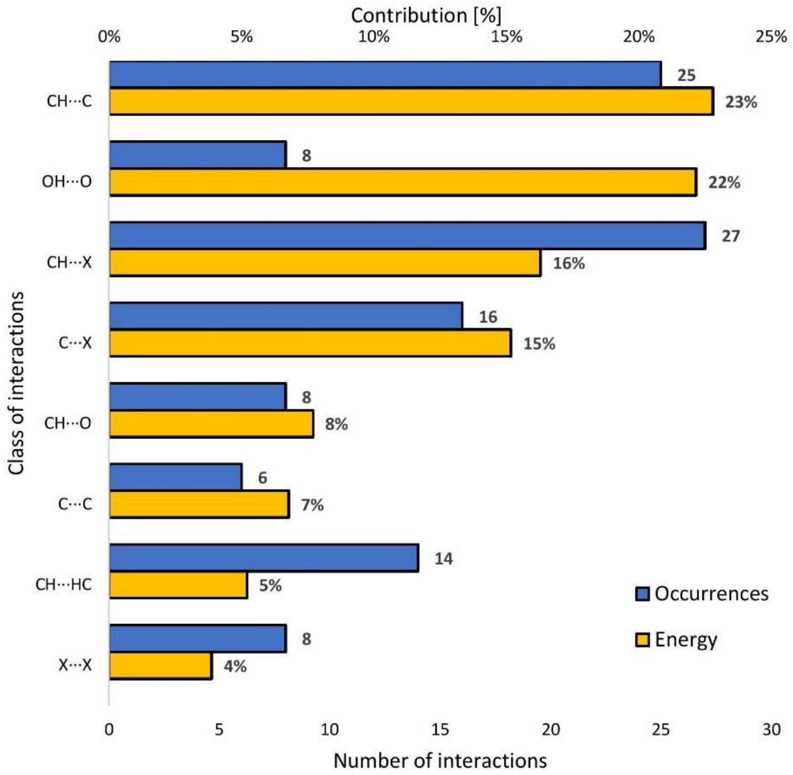

Figure S5. Number of occurrences and energetic contribution of each class of intermolecular interactions for all compounds.

To investigate the conformity of the interaction energy values found and validate the approach that will be used in the next stage of working out crystallization mechanisms, we compared the lattice energy(LE) [32] data with the melting points of the compounds studied. The lattice energy for a single crystal is defined as the difference between the internal energy calculated for a mole of constituent molecules in infinite separation and the energy for a mole of the same molecules in the crystalline state. In practical terms, the lattice energy represents the sum of all the different intermolecular interactions present in the first coordination sphere. The melting point, in turn, is the temperature at which a substance changes from a solid to a liquid state. Intermolecular interactions are fundamental to interpreting phenomena that affect the physical-chemical properties of substances, such as the melting point. The stronger the intermolecular interactions, the more energy must be supplied to break these interactions and cause the substance to change physical state. Thus, in most cases, lattice energy and melting point are directly proportional if the compounds are similar.

Table S4. Lattice energy and melting point of halogenated benzyl alcohols.

Compound	Lattice energy (kcal.mol^{-1})	Melting point (°C)[48]
o-Cl	20,57	69-71
o-Br	21,75	78-80
p-Cl	20,39	68-71
p-Br	21,20	75-77

In studying the compounds, a clear trend was observed: as the atomic radius increases (Cl < Br), both the melting point and the calculated lattice energy also increase. This trend demonstrates the importance of the atomic contact surface in determining the energy of intermolecular interactions.[32] The relationship observed, where higher EL values correspond to higher melting points, is in line with the fact that stronger intermolecular interactions require more energy to be overcome, allowing the change of physical state. Based on the correlation obtained between the theoretical and experimental data, we consider that the energy values are appropriate to continue with the study and start formulating proposals for the crystallization mechanisms of organic structures.

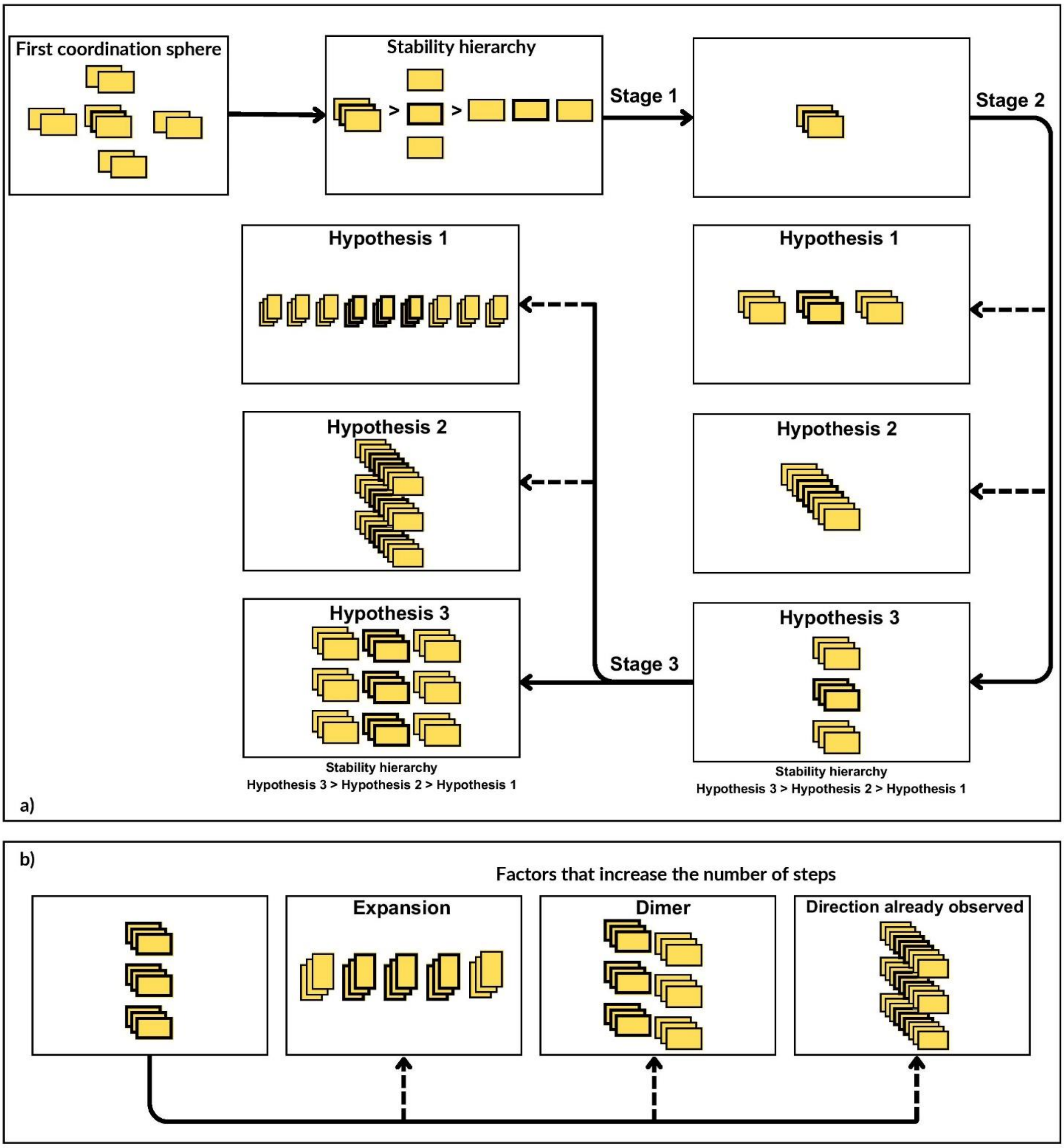

Figure S6. Generic demonstration of the application of the model a) Conventional crystallization route; b) Factors influencing the number of steps.

For the p-Br compound, the first step occurs by C-H···π and Br···π interactions by approximations from both sides (Figure S7) of a central molecule, thus generating a chain of three molecules along the b axis. This interaction presented a contact area of 23.78 Å^2 and an interaction energy of -5.80 kcal.mol^{-1}. For step 2, a dimeric interaction between chains is observed through the O···H-O interaction, with a contact area of 48.42 Å^2 and an energy of -20.41 kcal.mol^{-1}. Step 3 is motivated by C-H···H-C, C-H···Br and C-H···O interactions that form a supramolecular layer along the *a* axis by the approach of supramolecular structures to the central structure, with a contact area of 231.83 Å^2 and energy of -40.94 kcal.mol^{-1}. For the next step (step 4) the expansion of the previous step was observed. For step 5 a new π stacking was verified, from C-H···π and Br···π

interactions with a contact area of 454.76 $Å^2$ and interaction energy of -110.63 $kcal.mol^{-1}$ indicating a growth along the b axis. Finally, C-H···Br and Br···Br interactions guide growth along the c axis in step 6, with a contact area of 1666.09 $Å^2$ and energy of -340.62 kcal.mol-1 and growth along the c-axis (Figure S7). This concludes the proposed crystallization mechanism of p-Br benzyl alcohol.

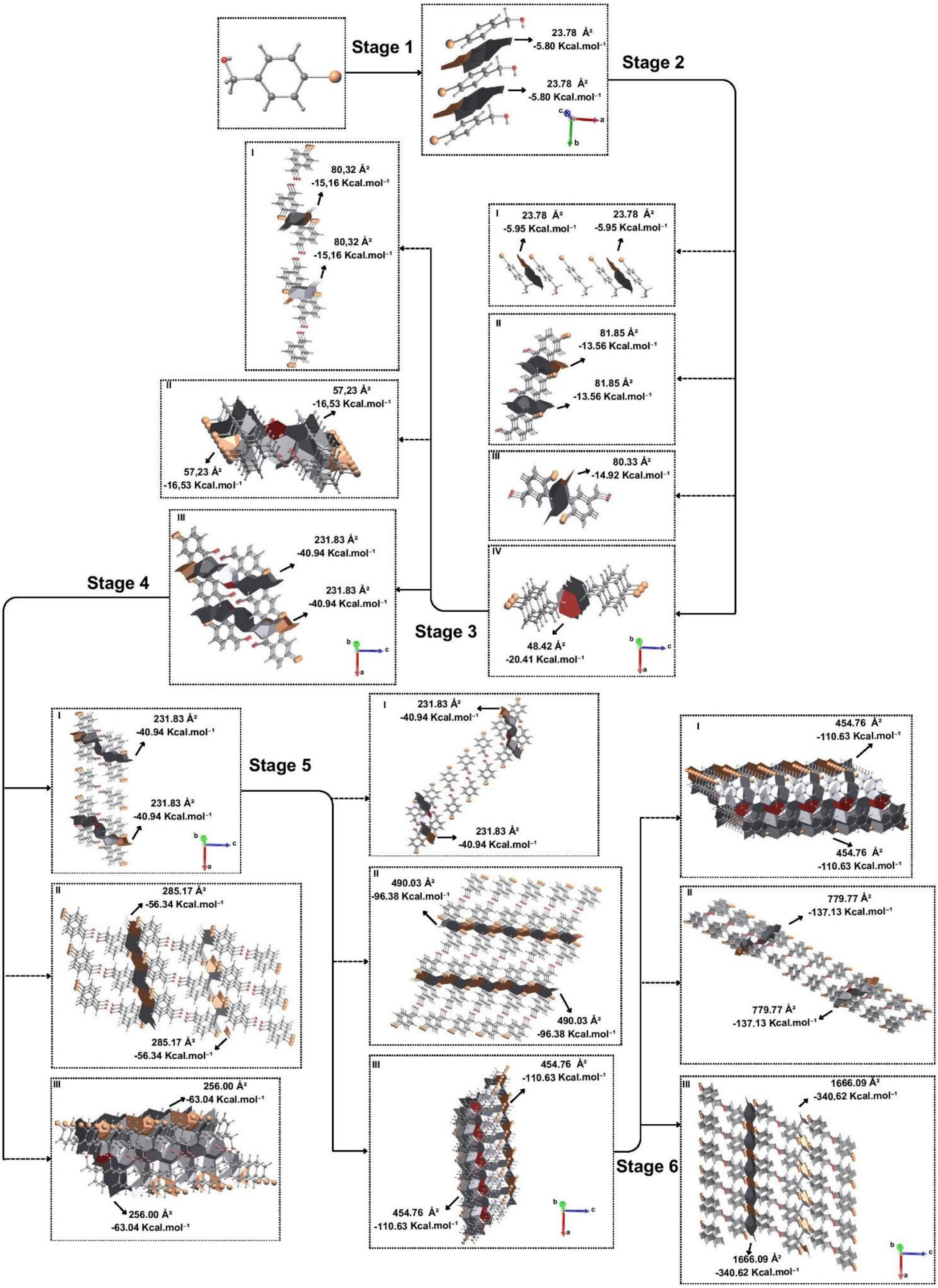

Figure S7. Proposed crystallization mechanism for p-Bromo substituted benzyl alcohol.

References

[48] Sigma-Aldrich Co., *2012-2014 Sigma-Aldrich catalog: Handbook of fine chemicals,* Aldrich Chemical*,* **2011**.
